# The effectiveness of exercise on the symptoms in breast cancer patients undergoing adjuvant treatment: an umbrella review of systematic reviews and meta-analyses

**DOI:** 10.3389/fonc.2023.1222947

**Published:** 2023-09-20

**Authors:** Yuan Zhao, Leiwen Tang, Jing Shao, Dandan Chen, Yueying Jiang, Panpan Tang, Xueqing Wang

**Affiliations:** ^1^ Department of Nursing, Sir Run Run Shaw Hospital, Zhejiang University, School of Medicine, Hangzhou, Zhejiang, China; ^2^ School of Medicine, Zhejiang University, Hangzhou, Zhejiang, China

**Keywords:** exercise, breast cancer, adjuvant treatment, symptom, umbrella review

## Abstract

**Background:**

Exercise has the potential to reduce symptoms for breast cancer patients during adjuvant treatment, and high-quality systematic reviews are essential for guiding clinical practice. The objective of this umbrella review is to examine current research evidence concerning the effectiveness of exercise on symptom management in breast cancer patients undergoing adjuvant treatment.

**Methods:**

An umbrella review was conducted. We searched for eligible systematic reviews through 11 databases until August 13^rd^, 2023. Two authors independently screened titles and abstracts, assessing the full-text studies based on inclusion criteria. We used AMSTAR-2 to appraise the quality of the meta-analyses. The results would be presented with narrative summaries if the replication rate of the original study for a symptom was higher than 5% (calculated via the Corrected Covered Area, CCA). The protocol was documented in the PROSPERO registry (CRD42023403990).

**Results:**

Of the 807 systematic reviews identified, 15 met the inclusion criteria, and 7 symptoms were the main focus. The main form of exercise mentioned was aerobic combined resistance exercise. The results of the quality assessment were mostly critically low (10/15). The repetition rate calculated by CCA showed moderate to very high repetition rates (10% to 18.6%). The findings of the included reviews indicated that the effects of exercise on relieving symptoms during breast cancer adjuvant treatment were mixed.

**Conclusions:**

Research is still needed to confirm the majority of studies’ recommendations for exercise during adjuvant treatment for breast cancer patients, as it is crucial for managing symptoms in the rehabilitation process. To increase the efficiency of exercise in symptom management, future studies may focus more on the application of bridge symptoms, symptom networks, and ecological instantaneous assessment.

## Introduction

According to the 2020 Global Cancer Statistics (1), female breast cancer has surpassed lung cancer as the most commonly diagnosed cancer, with an estimated 2.3 million new cases (11.7%) and 685000 deaths, making it already the fifth leading cause of cancer mortality worldwide. Breast cancer is the most common cancer among women, accounting for one in four new cases and one in six cancer-related deaths worldwide (1). In China, breast cancer makes up 15% of all new cancer cases in women, and it is the primary reason for cancer deaths in those under 45 (2).

With the improvements in early screening and treatment technologies, the survival period of breast cancer patients is prolonged. The need to improve the quality of life during cancer rehabilitation is becoming an increasingly important research topic with the rising number of survivors, particularly in breast cancer, the most common malignancy among women (3). According to the most recent statistics from the US, there are more than 4 million breast cancer survivors, including those who are still receiving treatment and those who have finished (4). This suggested that many breast cancer survivors must deal with persistently unpleasant side effects brought on by cyclical or long-term therapies such as chemotherapy, hormonal therapy, and radiation therapy (5). The majority of cancer therapies are assessed for safety based on acute side effects rather than chronic conditions that, while less serious, can have a considerable impact on the survivors’ quality of life for years to come (6). Oncology patients frequently only receive follow-up care that assesses tumor recurrence rather than treating other problems that are occasionally even overlooked. One of these easily overlooked problems is symptoms, which are also the main clinical manifestation of the adverse outcomes of the neoplasm or its therapy (7). Fatigue, sleeplessness, nausea, and vomiting are typical side effects of chemotherapy which often appear in the early stages of the chemotherapy cycle and may change afterward (8). Common and known side effects derived from post-chemotherapy hormone therapies, such as those based on aromatase inhibitors (AIs) and tamoxifen, may result in musculoskeletal, vasomotor, and urinary symptoms (9). Radiation therapy may bring symptoms like lymphedema, and cardiotoxicity (10, 11). Whether concurrent or not, most breast cancer patients experience these adjuvant treatments, as well as the distressing side effects associated with the treatment process, which can significantly impact patient outcomes.

Patients with breast cancer undergo not only medication-assisted treatment, or radiation therapy but also lifestyle and mental modifications as part of their rehabilitation process (12). Exercise oncology during this rehabilitation is receiving more and more attention (13, 14). Quality of life and physical activity levels during breast cancer rehabilitation are highly associated. Exercise may help to improve the quality of life, treatment-related symptoms, adjuvant therapy completion rates, and even health outcomes of breast cancer patients (15–18), making it a crucial component of rehabilitation care. Exercise benefits breast cancer patients with fatigue (19), depression and anxiety (20), musculoskeletal symptoms linked to aromatase inhibitors (21), lymphedema (22, 23), and inflammation (5), according to several studies. Some studies have also proven that exercise reduces the risk of developing other chronic diseases in breast cancer patients (24, 25).

Different exercise programs have different types, intensities, durations, and frequencies. When considering the type and dose of exercise, a multicenter study suggested that a higher volume of aerobic exercise or combined exercise may prevent the deterioration of symptoms related to adjuvant therapy (26). Another trial showed that supervised, moderate- to high-intensity resistance and aerobic exercise programs were most effective in reducing fatigue, musculoskeletal, and gastrointestinal symptoms for breast cancer patients receiving chemotherapy (17). And the combination of exercise with whole-body vibration might be considered a secure and well-tolerated intervention to reduce musculoskeletal symptoms, which was revealed in a pilot study (27). Recently, a randomized controlled study (28) examined the effects of various exercise doses on patient-reported outcomes in breast cancer patients during chemotherapy. But the trend plots in this study for various symptoms over time revealed no absolute association between exercise dose and symptoms. With regard to the phase of the exercise, some trials explored exercise programs during or after adjuvant therapy for breast cancer (29, 30), other researchers indicated that exercise should begin early in the diagnosis of breast cancer (31, 32), which might help to achieve the exercise goal at the end of adjuvant therapy and also made it easier to maintain exercise during rehabilitation after discharge. In terms of the duration of exercise, most studies followed up for a short term, which was within 6 months (33, 34).

There is still work to be done in successfully transferring exercise oncology into clinical practice (35) since various breast cancer patients have distinct exercise demands and require different exercise programs. Given the side effects of breast cancer adjuvant treatments and the benefits of exercise in reducing treatment-related symptoms, current evidence concerning the effectiveness of exercise on symptoms among breast cancer patients receiving adjuvant treatment requires being thoroughly summarized through Umbrella Review. The characteristics of exercise interventions, the adjuvant treatments that breast cancer survivors received, and the symptoms they underwent are the major items we concentrate on and extract.

## Methods and materials

### Registration and literature search

This umbrella review protocol was documented in the PROSPERO registry (CRD42023403990).

We used JBI’s three-step process to conduct a more thorough search of the relevant articles. First, we conducted a limited search in two databases (PubMed and CINAHL), and then we examined the text words in the titles and abstracts of the papers we found. Up until December 8^th^, 2022, we conducted additional searches in databases, namely PubMed, Web of Science (WOS), the Cochrane Library, the Cumulative Index of Nursing and Allied Health (CINAHL) Plus with full text, Excerpta Medica dataBASE (EMBASE), PsycINFO, Wanfang Data Knowledge Service Platform, China National Knowledge Infrastructure (CNKI), China Biomedical Literature Database (CBMdisc), China Science and Technology Journal Database (VIP). With regard to the five main themes of “breast cancer,” “adjuvant treatment,” “exercise,” “symptoms,” and “systematic review,” we employed Medical Subject Headings (MeSH) terms and free texts based on PICOS (please see **Table 1** for the precise search strategies). We manually searched the references of the articles we chose to find additional sources. All acquired articles are saved in NoteExpress. We conducted a search update on August 13^rd^, 2023, and added searches in Scopus and the Cochrane Central Register of Controlled Trials (CENTRAL) in the Cochrane Library.

**Table 1:** Search strategies.

Database	Strategies and Results
PubMed	(((((((((((breast neoplasms[MeSH Terms]) OR (“breast neoplasm*”[Title/Abstract])) OR (“breast cancer”[Title/Abstract])) OR (“breast carcinoma”[Title/Abstract])) OR (“breast malignant tumor”[Title/Abstract])) OR (“mammary cancer”[Title/Abstract])) OR (“mammary carcinoma”[Title/Abstract])) OR (“mammary neoplasm*”[Title/Abstract]))) AND (((((((((((((Drug Therapy[MeSH Terms]) OR (Therapeutic[MeSH Terms])) OR (“therap*”[Title/Abstract])) OR (“Drug Therapy”[Title/Abstract])) OR (“chemotherapy”[Title/Abstract])) OR (radiotherapy[MeSH Terms])) OR (“radiotherapy”[Title/Abstract])) OR (“treat*”[Title/Abstract])) OR (“cure”[Title/Abstract])) OR (“hormone therapy”[Title/Abstract])) OR (“Endocrine therapy”[Title/Abstract])) OR (“Adjuvant therapy”[Title/Abstract])))) AND ((((((((((((((((((((exercise[MeSH Terms]) OR (aerobic exercise[MeSH Terms])) OR (motor activity[MeSH Terms])) OR (sports[MeSH Terms])) OR (resistance training[MeSH Terms])) OR (dance therapies[MeSH Terms])) OR (dancing[MeSH Terms])) OR (yoga[MeSH Terms])) OR (walking[MeSH Terms])) OR (“exercise*”[Title/Abstract])) OR (“physical activit*”[Title/Abstract])) OR (“aerobic exercise*”[Title/Abstract])) OR (“motor activit*”[Title/Abstract])) OR (“sport*”[Title/Abstract])) OR (“resistance training”[Title/Abstract])) OR (“dance therap*”[Title/Abstract])) OR (“dancing”[Title/Abstract])) OR (“yoga”[Title/Abstract])) OR (“walking”[Title/Abstract])))) AND ((((“symptom*”[Title/Abstract]) OR (“symptom cluster*”[Title/Abstract])) OR (“multiple symptoms”[Title/Abstract]))) Filters: Meta-Analysis, Systematic Review 65
Web of Science	#1 TS=(“breast neoplasm$” or “breast cancer” or “breast carcinoma” or “breast malignant tumor” or “mammary cancer” or “mammary carcinoma” or “mammary neoplasm$”) #2 TS=(therap* or “drug therapy” or “treatment$” or “chemotherapy” or “radiotherapy” or cure* or “hormone therapy” or “Endocrine therapy” or “Adjuvant therapy”) #3 TS=(exercise$ or “resistance training” or “motor activit*” or sport$ or “dance therap*” or dancing or yoga or walking or “physical activit*” or “aerobic exercise$”) #4 TS=(symptom$ or “symptom cluster$” or “multiple symptoms”) #5 #2 AND #1 AND #3 AND #4 #6 TS=(“systematic review” or “meta analysis” or meta-analysis) #7 #6 AND #5 258
Cochrane Library	#1 MeSH descriptor: [Breast Neoplasms] explode all trees 17939 #2 (“breast neoplasms” or “breast cancer” or “breast carcinoma” or “breast malignant tumor” or “mammary cancer” or “mammary carcinoma” or “mammary neoplasms”):ti,ab,kw 43502 #3 #1 or #2 43533 #4 MeSH descriptor: [Drug Therapy] explode all trees 180610 #5 MeSH descriptor: [Radiotherapy] explode all trees 10342 #6 therap* or “drug therapy” or treat* or “chemotherapy” or “radiotherapy” or “cure” or “hormone therapy” or “Endocrine therapy” or “Adjuvant therapy” 1318957 #7 #4 or #5 or #6 1333991 #8 #3 and #7 36193 #9 MeSH descriptor: [Exercise] explode all trees 38402 #10 MeSH descriptor: [Resistance Training] explode all trees 5529 #11 MeSH descriptor: [Motor Activity] explode all trees 41841 #12 MeSH descriptor: [Sports] explode all trees 20935 #13 MeSH descriptor: [Dance Therapy] explode all trees 125 #14 MeSH descriptor: [Dancing] explode all trees 286 #15 MeSH descriptor: [Yoga] explode all trees 1210 #16 MeSH descriptor: [Walking] explode all trees 8487 #17 (exercise or “resistance training” or “motor activity” or sports or “dance therapy” or dancing or yoga or walking or “physical activity” or “aerobic exercises”):ti,ab,kw 170423 #18 #9 or #10 or #11 or #12 or #13 or #14 or #15 or #16 or #17 174067 #19 (symptom or symptoms or “symptom cluster” or “multiple symptoms” or “symptom clusters”):ti,ab,kw 220492 #20 #8 and #18 and #19 679 #21 Cochrane Review 7
CINAHL, PsycINFO (from EBSCOhost)	TX (“breast neoplasms” or “breast cancer” or “breast carcinoma” or “breast malignant tumor” or “mammary cancer” or “mammary carcinoma” or “mammary neoplasms”) AND TX (“drug therapy” OR “treatment” OR “chemotherapy” OR “radiotherapy” OR “cure” OR “hormone therapy” OR “Endocrine therapy” OR “Adjuvant therapy”) AND TX (“exercise” or “resistance training” or “motor activity” or “sports” or “dance therapy” or “dancing” or “yoga” or “walking” or “physical activity” or “aerobic exercises”) AND TX (“symptoms” or “symptom cluster*” or “multiple symptoms”) AND TX (“systematic review” or “meta analysis” or “meta-analysis”) 10 (CINAHL)/12 (PsycINFO)
Embase	#1 ‘breast cancer’/exp OR ‘breast carcinoma’/exp OR ‘breast tumor’/exp OR ‘breast cancer’:ti,ab OR ‘breast carcinoma’:ti,ab OR ‘breast tumor’:ti,ab OR ‘breast neoplasm’:ti,ab OR ‘breast malignant tumor’:ti,ab OR ‘mammary cancer’:ti,ab OR ‘mammary carcinoma’:ti,ab OR ‘mammary neoplasm’:ti,ab #2 ‘exercise’/exp OR ‘aerobic exercise’/exp OR ‘motor activity’/exp OR ‘resistance training’/exp OR ‘sport’/exp OR ‘dance therapy’/exp OR ‘dancing’/exp OR ‘yoga’/exp OR ‘walking’/exp OR ‘physical activity’/exp OR ‘exercise’:ti,ab OR ‘aerobic exercise’:ti,ab OR ‘motor activity’:ti,ab OR ‘resistance training’:ti,ab OR ‘sport’:ti,ab OR ‘dance therapy’:ti,ab OR ‘dancing’:ti,ab OR ‘yoga’:ti,ab OR ‘walking’:ti,ab OR ‘physical activity’:ti,ab OR ‘motor activities’:ti,ab OR ‘dance therapies’:ti,ab OR ‘physical activities’:ti,ab #3 ‘drug therapy’/exp OR ‘chemotherapy’/exp OR ‘radiotherapy’/exp OR ‘therapy’/exp OR ‘hormonal therapy’/exp OR ‘cure’/exp OR ‘adjuvant therapy’/exp OR ‘drug therapy’:ti,ab OR ‘chemotherapy’:ti,ab OR ‘radiotherapy’:ti,ab OR ‘therapy’:ti,ab OR ‘hormonal therapy’:ti,ab OR ‘cure’:ti,ab OR ‘adjuvant therapy’:ti,ab OR ‘treatment’:ti,ab OR ‘endocrine therapy’:ti,ab #4 ‘symptoms’/exp OR ‘symptom cluster’/exp OR ‘multiple symptoms’:ti,ab OR ‘symptoms’:ti,ab OR ‘symptom cluster’:ti,ab #5 #1 AND #2 AND #3 AND #4 #5 AND (‘meta analysis’/de OR ‘systematic review’/de OR ‘systematic review topic’/de) 189
Scopus	((((TITLE-ABS-KEY(“breast neoplasm$” or “breast cancer” or “breast carcinoma” or “breast malignant tumor” or “mammary cancer” or “mammary carcinoma” or “mammary neoplasm$”)) AND (TITLE-ABS-KEY(therap* or “drug therapy” or “treatment$” or “chemotherapy” or “radiotherapy” or cure* or “hormone therapy” or “Endocrine therapy” or “Adjuvant therapy”))) AND (TITLE-ABS-KEY(exercise$ or “resistance training” or “motor activit*” or sport$ or “dance therap*” or dancing or yoga or walking or “physical activit*” or “aerobic exercise$”))) AND (TITLE-ABS-KEY(symptom$ or “symptom cluster$” or “multiple symptoms”))) AND (TITLE-ABS-KEY(“systematic review” or “meta analysis” or meta-analysis)) 222
Wanfang Data	Subject: (Breast Cancer or Breast Malignancy or Breast Tumor or Breast Carcinoma) and Subject: (Treatment or Chemotherapy or Radiation Therapy or Hormone Therapy or Endocrine Therapy or Adjuvant Therapy) and Subject: (Symptom Cluster or Symptoms) and Subject: (Exercise or Training or Workout or Activity or Mobility or Dancing or Yoga or Walking) and Subject: (Systematic Review or Meta-analysis)
CNKI	Subject: (Breast Cancer or Breast Malignancy or Breast Tumor or Breast Carcinoma) AND Subject: (Treatment or Chemotherapy or Radiation Therapy or Hormone Therapy or Endocrine Therapy or Adjuvant Therapy) AND Subject: (Exercise or Training or Workout or Activity or Mobility or Dancing or Yoga or Walking) AND Subject: (Symptom Cluster or Symptoms) AND Subject: (Systematic Review or Meta-analysis)
VIP	Any field: (Breast Cancer or Breast Malignancy or Breast Tumor or Breast Carcinoma) AND Any field: (Treatment or Chemotherapy or Radiation Therapy or Hormone Therapy or Endocrine Therapy or Adjuvant Therapy) AND Any field: (Exercise or Training or Workout or Activity or Mobility or Dancing or Yoga or Walking) AND Any field: (Symptom Cluster or Symptoms) AND Any field: (Systematic Review or Meta-analysis )
CBM	(Breast Cancer[all fields:smart] OR Breast Malignancy[all fields:smart] OR Breast Tumor[all fields:smart] OR Breast Carcinoma[all fields:smart]) AND (Treatment[all fields:smart] OR Chemotherapy[all fields:smart] OR Radiotherapy"[all fields:smart] OR Hormone therapy[all fields:smart] OR Endocrine therapy[all fields:smart] OR Adjuvant therapy[all fields:smart]) AND ( Symptom cluster[common field:smart] OR Symptoms[common field:smart]) AND (Exercise[Common Fields:Intelligent] OR Training[Common Fields:Intelligent] OR Activity[Common Fields:Intelligent] OR Moving[Common Fields:Intelligent] OR Dancing[Common Fields:Intelligent] OR Yoga[Common Fields. Intelligent] OR Walking[Common Fields:Intelligent] AND (Systematic Review[Common Fields:Intelligent] OR Meta-analysis[Common Fields:Intelligent]

### Inclusion and exclusion criteria

The PICOS framework (Population, Intervention, Comparison, Outcome, Study design) was used for the inclusion and exclusion criteria for this literature: (1) the study population is breast cancer patients; (2) studies assess the effects of exercise on symptom management, with the exercise intervention spanning the period from initiation to the cessation of cancer adjuvant treatment; (3) the control group can be in any intervention; (4) studies measuring the symptoms as primary or secondary outcome indicators (these symptoms are measurable using symptom screening tools and may have a clinical impact on breast cancer patients, they may not always meet established diagnostic criteria.); (5) studies are meta-analyses conducted with systematic methods (both observational and interventional studies were available). Included studies required to outline at least one adjuvant treatment used on breast cancer patients. Due to the dearth of resources for translation into other languages, we only considered studies published in English or Chinese. Less than three studies or 100 individuals were not included. We chose the one that provided the most in-depth details in the repeated literature. The updated reviews were only included in their most recent iteration. Excluded items included books, editorials, comments, trial procedures, and conference papers.

### Study selection and data extraction

Two researchers independently evaluated the retrieved eligible articles by scanning the titles and abstracts of each study in accordance with the inclusion criteria after duplicates had been moved by NoteExpress. The remaining articles were then read in their entirety. Any disputes were settled through conversation or by requesting a third party.

The first author, publication year, country, guidelines, main databases, number of primary studies and participants, risk of bias, data synthesis, and main conclusion were among the data that two researchers independently extracted from the final eligible studies. We specifically extracted the characteristics of participants and data on how well exercise programs affected some symptoms. A file was used to hold all of the extracted data. Any discrepancies in the extracted data were discussed and resolved.

### Assessment of methodological quality of included studies

We used the AMSTAR-2 (A Measurement Tool to Assess Systematic Reviews version 2) (36) to assess the methodological quality of included meta-analyses. The 16 items in this measurement tool can each be responded to “yes”, “partial yes”, or “no”, among which seven items (items 2,4,7,9,11,13,15) are critical domains. AMSTAR-2 has shown good consistency, construct validity, and feasibility. Its overall reliability could be rated as “high” (no weakness or one noncritical item), “moderate” (more than one noncritical item), “low” (one critical item with or without noncritical items), or “critically low” (more than one critical item with or without noncritical items). Independent couples of two authors performed data extraction and the methodological assessment. In the same way, whenever there were any differences during this procedure, we talked about them or asked a third person for assistance.

### Method of analysis

The possibility of duplicate primary studies often exists in meta-analyses of similar topics. Current guidelines to address overlap suggest that assessing and documenting the degree of overlap in primary studies, calculated via the Corrected Covered Area (CCA) is a promising method (37). The calculation formula is “CCA=(N-r)/(r*c-r)”. (c=Number of included reviews, r=Number of publications of primary studies, N=Number of total primary studies including double counting). The calculation results could be slight (CCA<5%), moderate (CCA from 6% to 10%), high (CCA from 11% to 15%), and very high overlap (CCA>15%). We quantitatively pooled the final effect sizes when a certain symptom was estimated with a low repetition rate (less than 5%); otherwise, we would narratively summarize our findings. We provide a specific calculation of the repetition rate in **Supplementary Material 5**.

## Results

### Study selection

**Figure 1:**
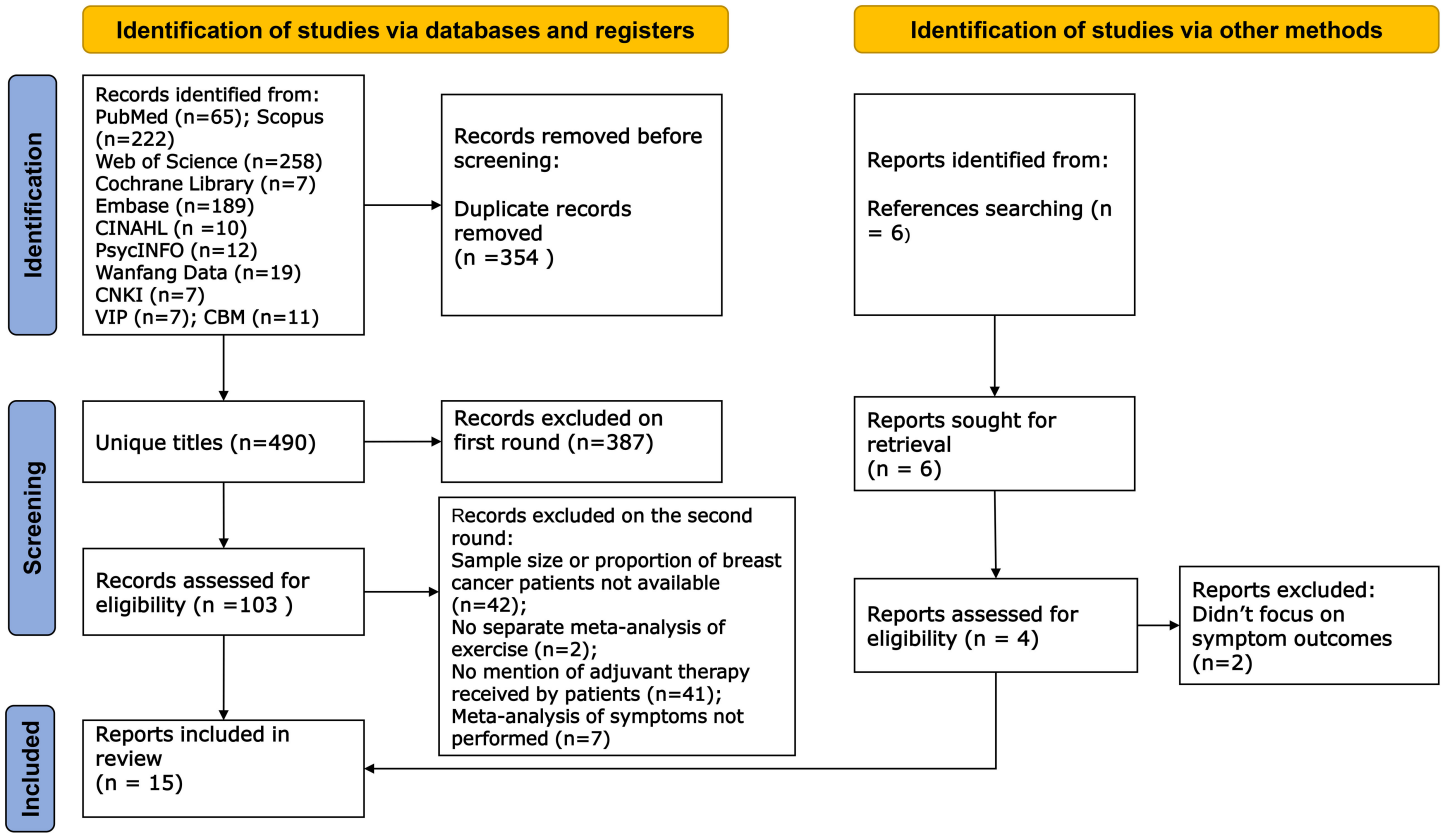
PRISMA diagram.

The literature search revealed 807 records, of which 317 were duplicates. After screening titles and abstracts, we excluded 387 reports on the first round: 294 studies were irrelevant to the theme; 36 had other interventions or included multiple interventions; 32 studies had ineligible literature types (narrative review, integrated review, umbrella review, or overview, scoping review); 16 studies without symptom outcomes; 7 protocols; 1 study was unavailable; 1 conference abstract; 103 reports remained. We conducted a second round of selection by reading the full text. In the end, we included 15 articles, of which 3 were Cochrane reviews. A PRISMA diagram is shown in **Figure 1**.

### The basic characteristics of the included studies

We summarized the main characteristics of the 15 included reviews in **Tables 2**, **3**. The 15 included reviews were conducted by Chinese (21, 41, 46, 48, 49) (n=5), German (40, 42)(n=2), Dutch (38) (n=1), American (45) (n=1), Brazil (51) (n=1), Australian (47) (n=1), Spanish (50) (n=1), Colombia (39) (n=1), Ireland (44) (n=1), and Greek (43) (n=1). The sample size included in meta-analyses varied with a wide range of 400 to 2964. The number of primary studies included in each systematic review ranged from 5 to 32. The included reviews were published in 2015 or later. All primary studies in eligible meta-analyses conducted randomized controlled trials, and the majority of them assessed the outcomes of the symptoms using self-reporting questionnaires. While publishing bias and reporting bias were rarely presented in studies.

**Table 2:** Characteristics of the reviews.

Study	Country	Guideline	Main databases	N of primary studies	N of participants	Risk of Bias	Data synthesis	Main conclusions								
Jonna K. vanVulpen, 2016 (38)	Netherlands	PRISMA	PubMed, Embase, Cochrane Library in June 2015	5 RCTs	784	Cochrane’s risk of bias tool	Random-effects model	General fatigue **＋**			Physical fatigue **＋**		Affective fatigue **〇**			
José Francisco Meneses-Echávez, 2015 (39)	Colombia	PRISMA	PubMed/ MEDLINE, EMBASE, Scopus, CENTRAL, CINAHL between December 2013 and January 2014	9 RCTs	1156	PEDro scale	Random-effects model	CRF								
								Supervised exercise **＋**				High-volume exercises **＋**				
Furmaniak AC, 2016 (40)	Germany	PRISMA	CBCGSR, CENTRAL, MEDLINE, EMBASE, CINAHL, WHOICTRP, SPORTDiscus, SIGLE, ProQuest, PsycINFO, Clinical Trials up to March 30, 2015	32RCTs	2626	Cochrane’s risk of bias tool	Random-effects model	Fatigue **＋**			Depression **＋**		Depression and anxiety **＋**			
Yuanqing PAN, 2017 (41)	China	PRISMA	PubMed, EMBASE, Cochrane Library throughout June 2013	16 RCTs	930	Cochrane’s handbook 5.2 standards	Random-effects model	Fatigue **＋**	Depression **＋**			Anxiety **＋**			Gastrointestinal symptoms, pain **＋**	
Cramer H, 2017 (42)	Germany	PRISMA	CBCSR, MEDLINE, Embase, CENTRAL, IndMED, ICTRP, Clinical trials up to January29, 2016	24RCTs	2166	Cochrane’s risk of bias tool	Random-effects model	Compared with no therapy				Compared with psychosocial or educational interventions				
								Fatigue **＋**				Depression **＋**				
								Sleep disturbances **＋**				Anxiety **＋**				
												Fatigue **＋**				
Patsou ED, 2017 (43)	Greece	PEDro criteria list	PubMed, Elsevier, Google Scholar between January, 2011 and November, 2016	14RCTs	1701	PEDro scale	Fixed-effects model	Depression **＋**								
Andrea Lipsett, 2017 (44)	Ireland	PRISMA	MEDLINE, EMBASE, Google Scholar, CINAHL, AMED and Clinical Trials Up to October 31, 2015	9RCTs	802	PEDro scale	Random-effects model	Fatigue **＋**								
Geling Lu, 2020 (21)	China	PRISMA	PubMed, CINAHL, EMBASE, VIP, WOS, Wan Fang, CNKI, CBM up to May 2019	9RCTs	743	Cochrane’s handbook	Random-effects model	Musculoskeletal symptom **＋**								
Ehlers DK, 2020 (45)	USA	PRISMA	PubMed, CancerLit, CENTRAL, PsychINFO, Clinical Trials, Breast Cancer Trials in March 2011 through April 2019	21RCTs	2588	Cochrane’s handbook and CLEAR checklist	Random-effects model	CRF **＋**								
Li-Juan Yi, 2021 (46)	China	PRISMA	PubMed, EMBASE, CNETRAL, CINAHL, CBM, VIP, CNKI, Wang Fang before September 2020	7RCTs	693	Cochrane’s handbook	Fixed-effects model and random-effects model			Short-term				Medium- and long-term		
								Fatigue		**＋**				**〇**		
								Depression		**＋**				**〇**		
								Anxiety		**＋**				**〇**		
								Sleep disturbance		**＋**				**〇**		
								Qol		**＋**				**〇**		
Roberts KE, 2020 (47)	Australia	PRISMA	CBCSR, CENTRAL, MEDLINE, Embase, CINAHL up to December 13, 2018	7RCTs	400	Cochrane’s risk of bias tool	Random-effects model and fixed-effects model	AIMSS **〇**								
Er Jung Hsueh, 2021 (48)	Taiwan	PRISMA	PubMed, Embase, Cochrane Library published before July 2020	26RCTs	2069	Cochrane’s handbook	Random-effects model	Fatigue **＋**	Depression **＋**			Anxiety **＋**	Sleep disturbance **＋**			Pain **＋**
Yang Yuan, 2022 (49)	China	PRISMA	CENTRAL, MEDLINE, Embase, WOS, CBM, Wanfang, VIP, CNKI from inception to March 2021	8RCTs	764	Cochrane assessment tool	Random-effects model	CRF **＋**								
Reverte-Pagola G, 2022 (50)	Spain	PRISMA	PubMed, CINAHL, WOS, CENTRAL, PsycINFO, EMBASE SportDiscus, Clinical Trials, until June 29, 2022	31RCTs	2964	Cochrane’s risk of bias tool	Random-effects model	Supervised				Non-supervised				
								Fatigue **＋＋**				Fatigue **＋**				
Medeiros Torres D, 2022 (51)	Brazil	PRISMA	MEDLINE, WOS, CENTRAL, Embase, Virtual Health Library Portal until June 22, 2020	20RCTs	1793	PEDro scale	Random-effects model	Fatigue **＋**								

Cumulative Index of Nursing and Allied Health (CINAHL), Cochrane Breast Cancer’s Specialized Register(CBCSR), Cochrane Central Register of Controlled Trials (CENTRAL), Indexing of Indian Medical Journals (IndMED), International Clinical Trials Registry Platform (ICTRP), Cochrane Breast Cancer Group’s Specialized Register(CBCGSR), Physiotherapy Evidence Database (PEDro), World Health Organization International Clinical Trials Registry Platform (WHO ICTRP), SPORTDiscus, CancerLit, Breast Cancer Trials, China National Knowledge Infrastructure (CNKI), China Biomedical Literature Database (CBMdisc), Web of Science (WOS), Quality of life (Qol)

++: Greater effect.

+: Beneficial effect/effective.

〇: Unclear.

**Table 3:** Characteristics of the exercise.

Study	Treatment	Exercise intervention in studies			Symptoms				
		Types	Duration	Frequency	Fatigue	Depression	Anxiety	Sleep disturbances	Other symptoms
Jonna K. van Vulpen, 2016 (38)	During adjuvant treatment	Aerobic/resistance/combined	12 weeks to 18 weeks	Not summarized	✔				
José Francisco Meneses-Echávez, 2015 (39)	During active treatment	Aerobic training	Mean length of 21.4 weeks (SD 15.8)	Mean duration of 44.3 minutes (SD 15.2) Average of 2.5 (SD 0.7) sessions per week	✔				
Furmaniak AC, 2016 (40)	Receive adjuvant therapy	Aerobic, resistance, aerobic-resistance	6 weeks to 52 weeks	Not summarized	✔	✔	✔		Lymphoedema
Yuanqing PAN, 2017 (41)	Receive adjuvant radiation or chemotherapy	Yoga	3 weeks to 6 months	1-3 times a week, 60 to 90 minutes in length	✔	✔	✔		Gastrointestinal symptoms, pain
Cramer H, 2017 (49)	Receive active or complete treatment	Yoga	2 weeks to 6 months	1 to 10 times a week, 20 to 120 minutes in length	✔	✔	✔	✔	
Patsou ED, 2017 (43)	During and after treatment	Aerobic, resistance, aerobic and resistance and yoga exercises	6 weeks to 52 weeks	2 to 3 sessions per week, 30 to 90 minutes per session		✔			
Andrea Lipsett, 2017 (44)	During adjuvant radiotherapy	Resistance training, combined aerobic-resistance exercise, low-intensity mind-body exercise, home-based aerobic exercise	5 weeks to 12 weeks	2 to 5 times per week, 10-60 minutes a time	✔				
Geling Lu, 2020 (21)	Taking aromatase inhibitors	Aerobic exercise (walking, aquatic exercise, strength training, bench press, leg press, seated row and so on)	6 weeks to 12 months	At least 120 minutes a week					AIMSS
Ehlers DK, 2020 (45)	During active treatment	Aerobic, combination, or mind-body exercise	3 weeks to 27 weeks	30 to 60 minutes per session, 3 or more days per week	✔				
Li-Juan Yi, 2021 (46)	Receiving chemotherapy	Yoga (Tibetan, Bali, Anusara, Dru, integrated yoga programs and so on)	8 weeks to 4 months	1 to 4 times a week, 50- to 90-minute sessions	✔	✔	✔	✔	
Roberts KE, 2020 (47)	hormone therapy with AIs	Aerobic/resistance/combined/other	6 weeks to 12 months	At least 150 to 200 minutes weekly					AIMSS
Er Jung Hsueh, 2021 (48)	Undergoing breast cancer treatment	Yoga practice (body posture practices, breathing exercises, and meditation)	1 week to 12 weeks	Each session lasting 60 to 120 min	✔	✔	✔	✔	Pain
Yang Yuan, 2022 (49)	Receiving multiple anticancer treatments	Home-Based Walking	6 weeks to 6 months	2 to 6 times per week, 20 to 40 minutes a time	✔				
Reverte-Pagola G, 2022 (50)	During or after adjuvant treatments	Supervised (endurance, resistance or combined, yoga, hydrotherapy) or Non-supervised exercise (endurance, resistance or combined, yoga, Nia)	4 weeks to 32 weeks	2 to 7 times a week 10 to 90 minutes in length	✔				
Medeiros Torres D, 2022 (51)	During adjuvant chemotherapy, radiotherapy	Supervised combination of resistance training (RT) with aerobic training (AT), mind–body techniques.	6 to 24 weeks	1-6 times a week, 7 to 75 minutes a time	✔				

Aromatase inhibitor-induced musculoskeletal symptoms (AIMSS).

Among 15 systematic reviews, 9 involved multimodal exercise (21, 38, 40, 43–45, 47, 50, 51), which could be a combination of aerobic and resistance exercise (21, 38, 40, 43–45, 47, 51), or a combination of supervised and unsupervised exercise (50). Yoga was mentioned in 4 systematic reviews (41, 42, 46, 48). 1 systematic review concentrated on aerobic exercise (39), and 1 focused on home-based walking (49). The total duration of the exercise interventions was between 1 week to 52 weeks, with most of them lasting 3 weeks to 6 months. Short-term exercise is considered an exercise intervention within 6 months, 6~12 months belongs to the medium-term, and exercise beyond 12 months is long-term (46). Most original studies contained in the included reviews (38, 39, 41, 42, 44, 46, 48, 49, 51) had a follow-up time of 6 months, with insufficient evidence of medium- and long-term exercise. Most systematic reviews (11/15) described the exercise frequency by providing the number of times per week and the duration of each exercise (39, 41–46, 48–51), and some described the total duration of one week’s exercise (21, 47). 2 reviews didn’t generally summarize the exercise frequency (38, 40). In all included systematic reviews, the effects of exercise on a total of seven symptoms—fatigue, anxiety, depression, sleep disturbance, musculoskeletal symptoms, lymphedema, and gastrointestinal symptoms—were examined.

### Effectiveness of exercises on fatigue symptoms

Please see **Table 3** for details on the effect of exercise on various symptoms and exercise-related characteristics. The effectiveness of exercises on fatigue symptoms in breast cancer patients was estimated by 18 meta-analyses from 12 systematic reviews (38–42, 44–46, 48–51) that were included. The final CCA result of 12.1% shows a significant number of primary reports that overlap. During adjuvant treatment for breast cancer patients, one review (38) found that exercise helped reduce general fatigue, notably physical fatigue, but had no effect on affective fatigue. Another review (50) found that supervised exercise had a greater anti-fatigue effect than unsupervised exercise. In breast cancer patients receiving adjuvant therapy, several systematic reviews concluded that short-term exercise (within 6 months) was effective in reducing fatigue (38, 39, 41, 42, 44, 48, 49, 51), although the effects of medium- and long-term exercise on reducing fatigue were erratic (40, 45, 46, 50).

### Effectiveness of exercises on depression and anxiety symptoms

Seven meta-analyses in total, chosen from six systematic reviews (40–43, 46, 48), were used to determine the effect of exercise on depression. A high repetition rate is indicated by the CCA, which is 14.4%. After eliminating duplicates from five systematic reviews (40–42, 46, 48) with 43 primary studies, anxiety was analyzed and the CCA was extremely high (18.6%). The findings of the systematic reviews showed that short-term exercise had a positive effect on relieving depression and anxiety in breast cancer patients during adjuvant treatment. One systematic review (46) compared the effectiveness of short-term exercise with medium- to long-term exercise in reducing depression and anxiety, and found that there was relatively insufficient evidence for medium- to long-term exercise on both symptoms.

### Effectiveness of exercises on sleep disturbance symptoms

The effect of exercise on sleep disturbance symptoms in breast cancer patients was examined in six meta-analyses that were selected from three systematic reviews (40, 42, 46). 10% is the final CCA, which indicates a moderate duplicate rate. There are few research on the effects of exercise in reducing sleep disturbance in breast cancer patients receiving adjuvant therapy, and the medium- and long-term effects of exercise on sleep disturbance are unclear. Short-term exercise was recommended for reducing sleep disturbance during breast cancer adjuvant treatment according to the findings of all meta-analyses (42, 46, 48).

### Other symptoms findings

Two included reviews (21, 47) analyzed the effect of exercise in reducing musculoskeletal symptoms among women with breast cancer during the hormone therapy period (taking aromatase inhibitors). Three original studies were repeated in these two systematic reviews, leaving nine original studies. An inconsistent conclusion was reached by two systematic reviews. While one research found that exercise could successfully effectively relieve musculoskeletal symptoms and advised encouraging patients to exercise actively under medical supervision (21), another found no clear evidence of the effect of exercise (47). The HOPE study by Irwin et al (52). was the one contained in both of these two reviews. With the largest sample size among the primary studies (of 121 participants with breast cancer), the HOPE study reported a 29% improvement in pain symptoms assigned to exercise, compared to a 3% increase in those receiving usual care at 12 months.

Just one included meta-analysis (41) mentioned gastrointestinal symptoms such as nausea, vomiting, or loss of appetite, and the final aggregated estimation revealed a small effect with moderate heterogeneity (SMD= -0.39, 95%CI -0.54, -0.25, P= .00, I^2 = ^37.8%).

### Heterogeneity of included studies

Between the primary studies included in each meta-analysis, there were differences in a number of factors, including sophisticated exercise programs, various types of adjuvant therapies and symptoms, and how symptom outcomes were quantified, etc. Therefore, there is significant heterogeneity among the included systematic reviews and meta-analyses.

### AMSTAR 2 assessment results of the included studies

All of the studies with an overall confidence rating of “high” come from the Cochrane Library (40, 42, 47), whereas 10 studies have “critically low” ratings (21, 38, 39, 41, 43–46, 48, 49), and 2 studies have “low” ratings (50, 51). The essential items 2, 4, and 7 don’t match the standards, which is the leading cause of the low score. We find that 12 out of 15 studies have either a list of omitted studies or an explanation for exclusions (21, 38, 39, 41, 43–46, 48–51). In comparison, 8 studies didn’t register with procedures (21, 38, 41, 43–46, 49), 5 didn’t supply a complete retrieval strategy (21, 43, 45, 46, 48), and 3 have only provided a partial one (38, 39, 41). When evaluating the included 15 pieces of literature, the answers to items 1, 3, 8, 9, 11, 13, and 14 are all “yes” at 100%. **Table 4** contains the specifics of each evaluation item.

**Table 4:** The methodological quality of the included reviews according to the AMSTAR 2 tool.

	Cramer H 2017	Roberts KE 2020	Furmaniak AC 2016	Reverte-Pagola G 2022	Jonna K. van Vulpen 2016	Ehlers DK 2020	Geling Lu 2020	Yuanqing PAN2017	Patsou ED 2017	Li-Juan Yi 2021	Er Jung Hsueh 2021	José Francisco Meneses-Echávez 2015	Medeiros Torres D 2022	Andrea Lipsett 2017	Yang Yuan 2022
1. Did the research questions and inclusion criteria for the review include the components of PICO? Yes/No	Yes	Yes	Yes	Yes	Yes	Yes	Yes	Yes	Yes	Yes	Yes	Yes	Yes	Yes	Yes
**2. Did the report of the review contain an explicit statement that the review methods were established before the conduct of the review and did the report justify any significant deviations from the protocol?** **Yes/Partial Yes/No**	**Yes**	**Yes**	**Yes**	**Yes**	**No**	**No**	**No**	**No**	**No**	**No**	**Yes**	**Yes**	**Yes**	**No**	**No**
3. Did the review authors explain their selection of the study designs for inclusion in the review? Yes/No	Yes	Yes	Yes	Yes	Yes	Yes	Yes	Yes	Yes	Yes	Yes	Yes	Yes	Yes	Yes
**4. Did the review authors use a comprehensive literature search strategy?** **Yes/Partial Yes/No**	**Yes**	**Yes**	**Yes**	**Yes**	**Partial Yes**	**No**	**No**	**Partial Yes**	**No**	**No**	**No**	**Partial Yes**	**Yes**	**Yes**	**Partial Yes**
5. Did the review authors perform study selection in duplicate? Yes/No	Yes	Yes	Yes	Yes	Yes	Yes	Yes	Yes	No	Yes	No	Yes	Yes	Yes	Yes
6. Did the review authors perform data extraction in duplicate? Yes/No	Yes	Yes	Yes	Yes	Yes	Yes	Yes	Yes	No	Yes	Yes	Yes	Yes	Yes	Yes
**7. Did the review authors provide a list of excluded studies and justify the exclusions?** **Yes/Partial Yes/No**	**Yes**	**Yes**	**Yes**	**No**	**No**	**No**	**No**	**No**	**No**	**No**	**No**	**No**	**No**	**No**	**No**
8. Did the review authors describe the included studies in adequate detail? Yes/Partial Yes/No	Yes	Yes	Yes	Yes	Yes	Yes	Yes	Yes	Yes	Yes	Yes	Yes	Yes	Yes	Yes
**9. Did the review authors use a satisfactory technique for assessing the risk of bias (ROB) in individual studies that were included in the review?** **Yes/Partial Yes/No/Includes only NRSI/RCTs**	**Yes**	**Yes**	**Yes**	**Yes**	**Yes**	**Yes**	**Yes**	**Yes**	**Yes**	**Yes**	**Yes**	**Yes**	**Yes**	**Yes**	**Yes**
10. Did the review authors report on the sources of funding for the studies included in the review? Yes/No	Yes	Yes	Yes	Yes	Yes	No	No	No	No	No	Yes	No	No	No	No
**11. If meta-analysis was performed did the review authors use appropriate methods for the statistical combination of results?** **Yes/No/No meta-analysis (conducted)**	**Yes**	**Yes**	**Yes**	**Yes**	**Yes**	**Yes**	**Yes**	**Yes**	**Yes**	**Yes**	**Yes**	**Yes**	**Yes**	**Yes**	**Yes**
12. If meta-analysis was performed, did the review authors assess the potential impact of RoB in individual studies on the results of the meta-analysis or other evidence synthesis? Yes/No/No meta-analysis conducted	Yes	Yes	Yes	Yes	Yes	Yes	Yes	Yes	No	Yes	Yes	Yes	Yes	Yes	Yes
**13. Did the review authors account for RoB in individual studies when interpreting/discussing the results of the review? Yes/No**	**Yes**	**Yes**	**Yes**	**Yes**	**Yes**	**Yes**	**Yes**	**Yes**	**Yes**	**Yes**	**Yes**	**Yes**	**Yes**	**Yes**	**Yes**
14. Did the review authors provide a satisfactory explanation for, and discussion of, any heterogeneity observed in the results of the review? Yes/No	Yes	Yes	Yes	Yes	Yes	Yes	Yes	Yes	Yes	Yes	Yes	Yes	Yes	Yes	Yes
**15. If they performed quantitative synthesis did the review authors carry out an adequate investigation of publication bias (small study bias) and discuss its likely impact on the results of the review?** **Yes/No/No meta-analysis conducted**	**Yes**	**Yes**	**Yes**	**Yes**	**Yes**	**Yes**	**Yes**	**No**	**Yes**	**Yes**	**Yes**	**Yes**	**Yes**	**Yes**	**Yes**
16. Did the review authors report any potential sources of conflict of interest, including any funding they received for conducting the review? Yes/No	Yes	Yes	Yes	Yes	Yes	Yes	Yes	No	Yes	Yes	Yes	Yes	Yes	Yes	No
**Overall confidence**	**High**	**High**	**High**	**Low**	**Critically low**	**Critically low**	**Critically low**	**Critically low**	**Critically low**	**Critically low**	**Critically low**	**Critically low**	**Low**	**Critically low**	**Critically low**

## Discussion

### Effectiveness and problems of exercises on symptoms

To our knowledge, this is the first overview that thoroughly describes the role that exercise plays in symptom relief for breast cancer patients undergoing adjuvant treatment. Compared to previous umbrella reviews (53–55), which focused mainly on the benefits of exercise in reducing fatigue in cancer patients, we focused on breast cancer patients receiving adjuvant treatment and covered a wider range of symptoms. It is well known that during adjuvant treatment, the discomfort symptoms experienced by breast cancer patients typically appear in clusters, or “symptom clusters”, which refer to the simultaneous occurrence of at least two linked symptoms defined by Professor M. J. Dodd and other academics in 2001 (56). Symptoms are also interrelated and can be analyzed through symptom networks. Researchers presented the idea of “symptomics” by using symptom network analysis to study the particular link between symptoms (57), which further assisted in explaining the patterns of patient symptoms in the real world at the mechanistic level. It may contribute to increasing the efficiency of symptom management and performing targeted intervention with the novel perspective of assessing and analyzing the symptom cluster and symptom network of breast cancer patients during adjuvant treatment.

There were mixed effects of the exercise on fatigue according to the findings of our included systematic reviews. Short-term (within 6 months) exercise is effective in reducing general fatigue during adjuvant treatment in breast cancer patients, but the benefit of medium-term (6~12 months) or longer-term (more than 12 months) exercise is still unsure. Also, the effect of exercise on affective fatigue needs to be explored in more studies. Future studies should not only extend the duration of exercise interventions and follow-ups but also consider fatigue from a multidimensional perspective to precisely implement exercise interventions.

Our study’s comprehensive research revealed the alleviating effect of short-term exercise on anxiety and depression during adjuvant breast cancer treatment. Therefore, the medium- to long-term effects of exercise remain to be demonstrated. Furthermore, the relationship between mind and body may be taken into account, which may help to improve the effectiveness of exercise interventions. According to the relevant research, mindfulness yoga was an established mind-body exercise focused on mindfulness stress reduction (43). With good compliance in both groups (82.3% in the experimental group, 80.9% in the control group), a randomized controlled trial (58) found that mindfulness yoga was effective in reducing anxiety and depressive symptoms in breast cancer patients who had received adjuvant chemotherapy (anxiety inter-group effect 1.18, 95% CI: 0.2, 2.17; depression inter-group effect 1.49, 95% CI: 0.48, 2.5). Other mind-body exercises include Tai Chi and Qigong, both of which also have a lack of evidence in current studies for their effects in relieving anxiety or depression during adjuvant treatment of breast cancer. Based on this, future research could also explore whether mindfulness yoga, Tai Chi, or Qigong is more effective in improving symptoms during adjuvant breast cancer treatment compared to other exercises

Our final findings showed a positive effect of short-term exercise on sleep disturbance symptoms in three systematic reviews (40, 42, 46). The mechanism of correlation between symptoms uncovered by symptom clusters indicates that other unpleasant symptoms may contribute to or exacerbate sleep disturbances. One study in 2022 (59) put the symptoms (fatigue, sleep disturbance, and depression) as symptom clusters, which contained F-S-D (fatigue-sleep disturbance-depression), F-S (fatigue-sleep disturbance), F-D (fatigue-depression), and S-D (sleep disturbance-depression) symptom clusters. This study proposed a wide range of interventions that could alleviate all these four symptom clusters (F-S-D, F-S, F-D, S-D), further suggesting that symptom clusters may provide valuable clues for developing symptom management strategies, such as targeting an identified bridge symptom within or between core symptom clusters to improve the efficiency of the intervention.

In particular, aromatase inhibitors (AIs), which are linked to joint and muscular symptoms and are referred to as aromatase inhibitor-associated musculoskeletal symptoms (AIMSS) (60), are frequently used to treat hormone-positive (HR+) breast cancer in women. AIMSS are often an underdiagnosed and underestimated complication of such treatments. Data have shown that AIMSS can occur in up to 40% of HR+ breast cancer survivors receiving AI treatment (61), and that dropout rates for AIs due to the side effects can reach 20% during the first year of treatment (62–64). Bone health is always a challenge (12). The processes underlying AIMSS are still poorly understood, but they may be linked to extreme estrogen depletion (65). Previous studies have demonstrated the advantages of exercise in improving AIMSS in breast cancer patients by presenting pathological and physiological explanations: Exercise raises bone density (66), improves bodily fluid circulation to tissues (67), and may increase the pain threshold (68). However, our findings indicate that the empirical study of the effect of exercise on AIMSS is still limited and that the inconsistent results of the included studies call for larger sample sizes and longer follow-up periods.

Other symptoms, such as lymphedema and gastrointestinal symptoms, are also symptoms that may occur or be present during adjuvant therapy in breast cancer patients. However, there is a limited number of studies on exercise during adjuvant therapy to improve these symptoms. In the case of lymphedema, the role of exercise on lymphedema has not reached a consistent conclusion in research. In addition to this, most studies (69, 70) have focused only on whether exercise increases the risk of developing lymphedema in breast cancer patients but not on the effect of exercise on relieving lymphedema during breast cancer adjuvant therapy. Our study focused on the latter and there may be some bias. The risk of lymphedema varies among breast cancer patients receiving different treatments, and in order to develop a more precise Exercise Plan, future studies should analyze the main causes of lymphedema based on the clinical context of breast cancer patients receiving treatment and follow up the effect of exercise during adjuvant therapy on lymphedema for a longer time. In patients with breast cancer receiving adjuvant therapy, particularly during chemotherapy, gastrointestinal symptoms (GIs) are frequent. The included systematic review’s findings (41) revealed that exercise had small effects on GIs, but the evidence is still insufficient and needs to be supplemented by more studies. GI symptoms like nausea, vomiting, and bloating are prevalent in symptom clusters, but those clusters often are inconsistent (71). A lack of appetite and malnutrition may result from these symptoms. In addition to necessary nutritional intervention which was the only focus in existing studies (72, 73), future studies might look into the potential effectiveness of combining exercise and dietary interventions in mitigating gastrointestinal symptoms.

In conclusion, during adjuvant treatment for breast cancer patients, as multiple symptoms always occur simultaneously, we should concentrate on the idea of symptom clusters and explore precise exercise interventions for bridging symptoms within or between symptom clusters. Besides, symptom networks are an extension and deepening of symptom clusters and can identify mechanisms of complex symptom interactions as well as potential targets for intervention in the real world. These two topics might be the subject of future research to boost symptom management efficiency.

### The quality of the methodology needs to be improved

Overall, the quality of the included studies needs to be improved. The systematic review should strictly follow the PRISMA process and reporting standards, which can be found in EQUATOR online. According to the results of our quality assessment, the following three areas need the most improvement: The systematic review should be preceded by a study protocol and registration, followed by a thorough database search and the provision of search strategies for each database. Lastly, provide a list of excluded studies and justify the exclusions.

### Limitations of this study

This article has the following limitations. Firstly, although the retrieval approach is not restricted to English or Chinese, we did include meta-analyses that were only published in these two languages, which might have a language bias. Secondly, non-peer-reviewed literature, systematic reviews without meta-analyses were excluded, as were reviews with less than 3 primary studies or 100 participants, all of which could have resulted in reporting bias. Thirdly, we did not perform subgroup analyses of different types of exercise or symptoms, only presented the results descriptively. The final concern is that the inclusion criteria for our literature were severely restricted, which may also result in some bias.

### Implications for future research

Several symptoms brought on by adjuvant treatment for breast cancer can be alleviated with exercise, but this benefit needs to be further demonstrated. First, future studies must increase the sample size. Second, although a growing number of recent studies have explored changes in symptom clusters or symptom networks over time during breast cancer adjuvant treatment, their clinical practice is inadequate. The complexity of the symptoms is one potential cause. To solve this problem, future research may use ecological transient assessment to dynamically assess symptoms. Thirdly, we found that there is still a lack of research on the effects of exercise on relieving symptom clusters or symptom networks during adjuvant therapy in breast cancer patients. We expect that further research will be conducted to examine the effects of exercise on improving bridge symptoms identified within or between symptom clusters and, in addition, advance the development of symptomics using symptom network analysis. This will improve the efficiency of symptom management and better meet the specific needs of more patients with breast cancer.

## Conclusion

The findings of this study offer some suggestions for further research by shedding light on the contribution of exercises to several symptoms in breast cancer patients receiving adjuvant treatment. Future studies might examine the effect of exercise on relieving bridge symptoms found within or between core symptom clusters during breast cancer adjuvant treatment. Alternatively, we may promote the application of symptom network analysis and make use of ecological instantaneous assessment to track changes in symptom networks dynamically.

## Author contributions

All authors listed have made a substantial, direct, and intellectual contribution to the work, and approved it for publication.
