# Supplementary material for: The effectiveness of exercise on the symptoms in breast cancer patients undergoing adjuvant treatment: an umbrella review of systematic reviews and meta-analyses
Source: Front Oncol. 2023 Sep 20;13:1222947. doi: 10.3389/fonc.2023.1222947 (PMC10548878; doi:10.3389/fonc.2023.1222947)

**Literature search results up to 2023.08.13**

**PubMed 65**


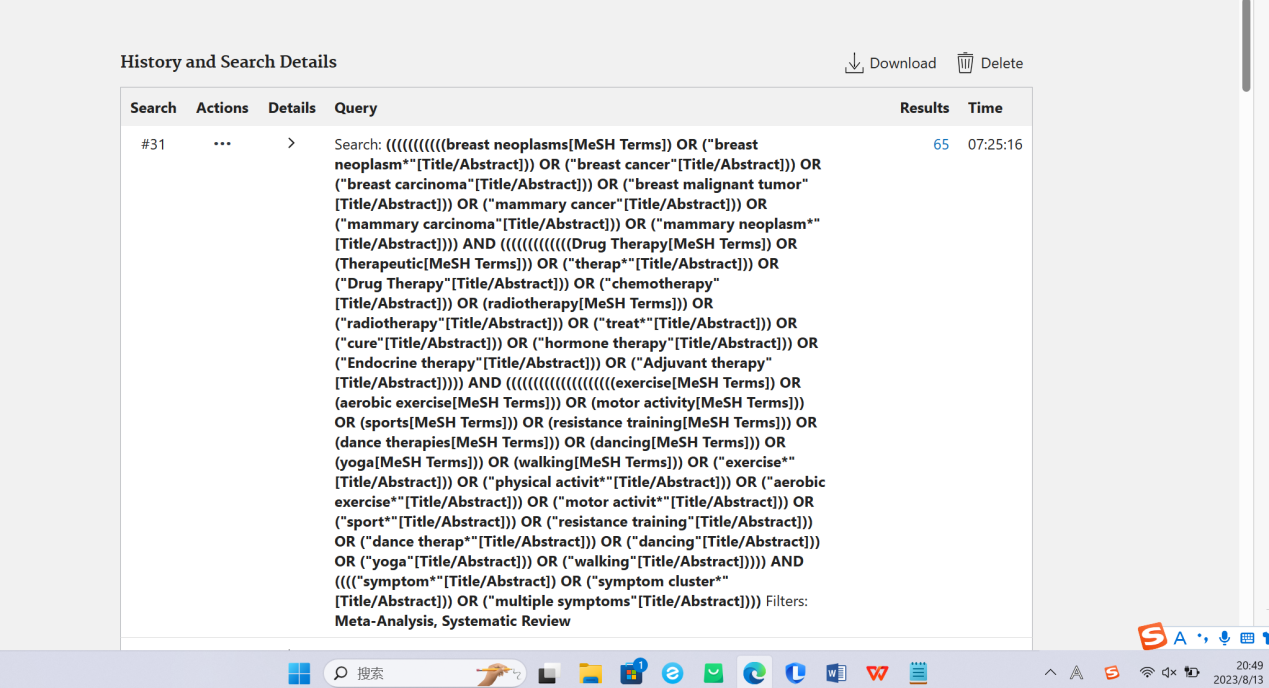


**Web of Science 258**


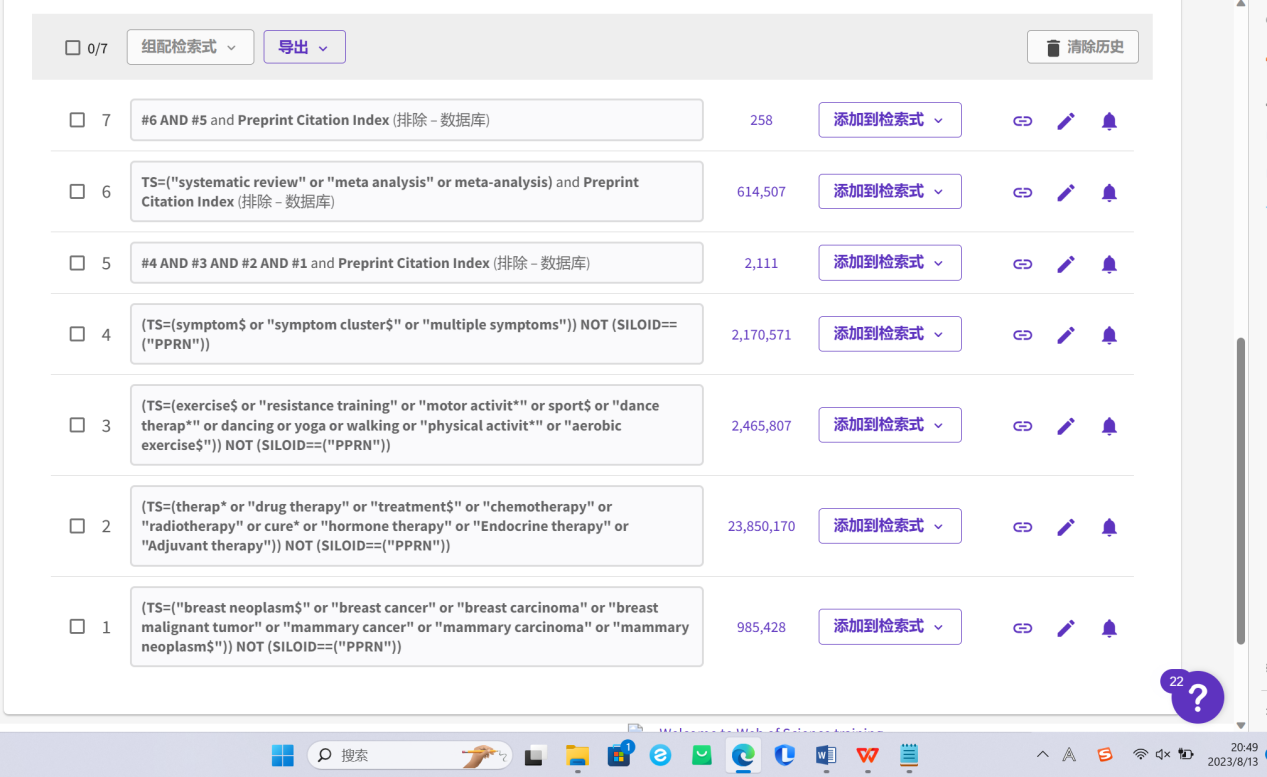


**Cochrane Library 7**


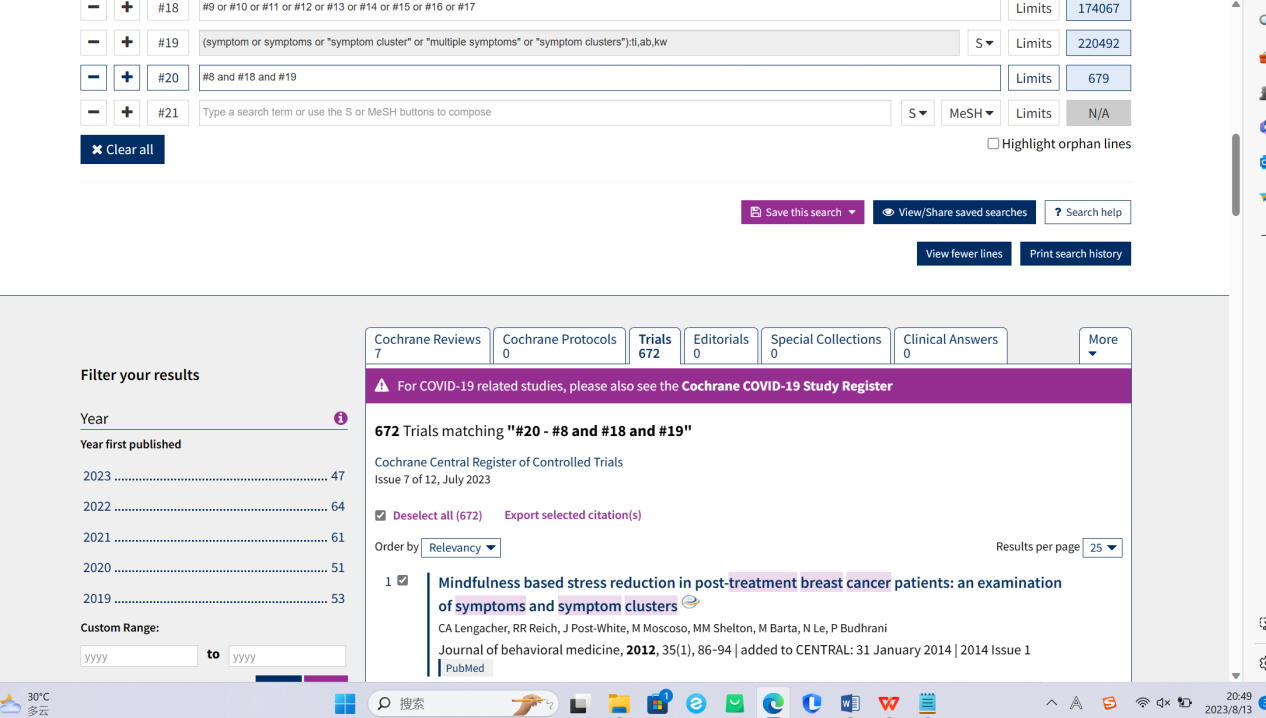


**Embase 189**


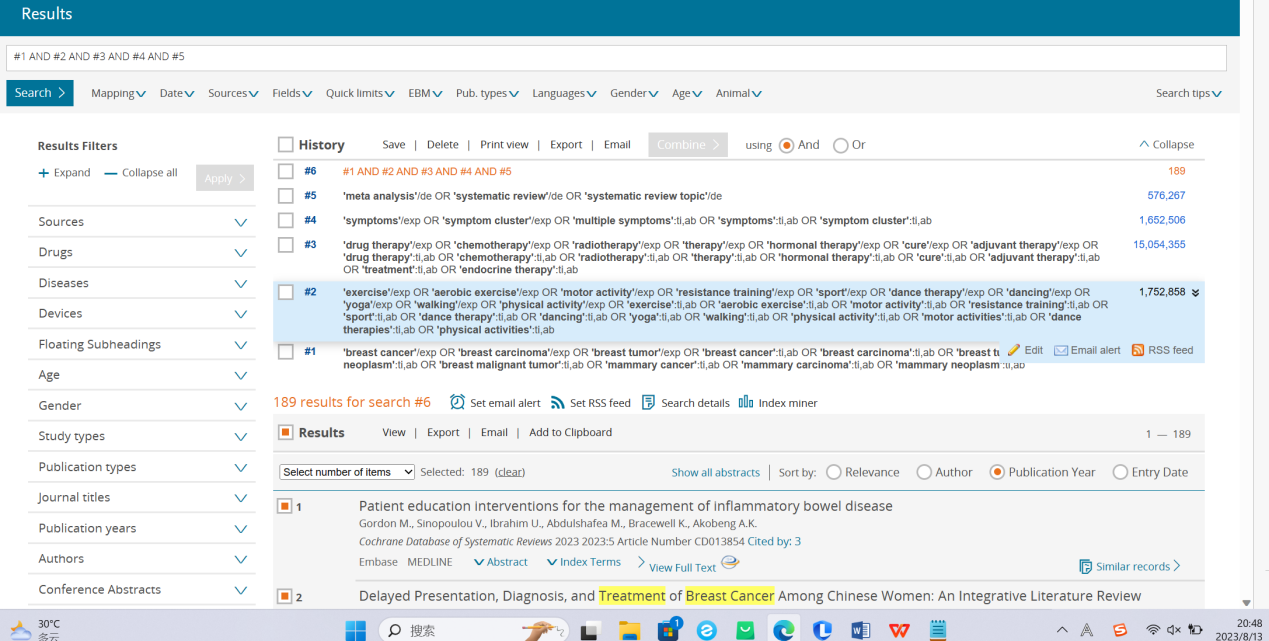


**CINAHL 10**


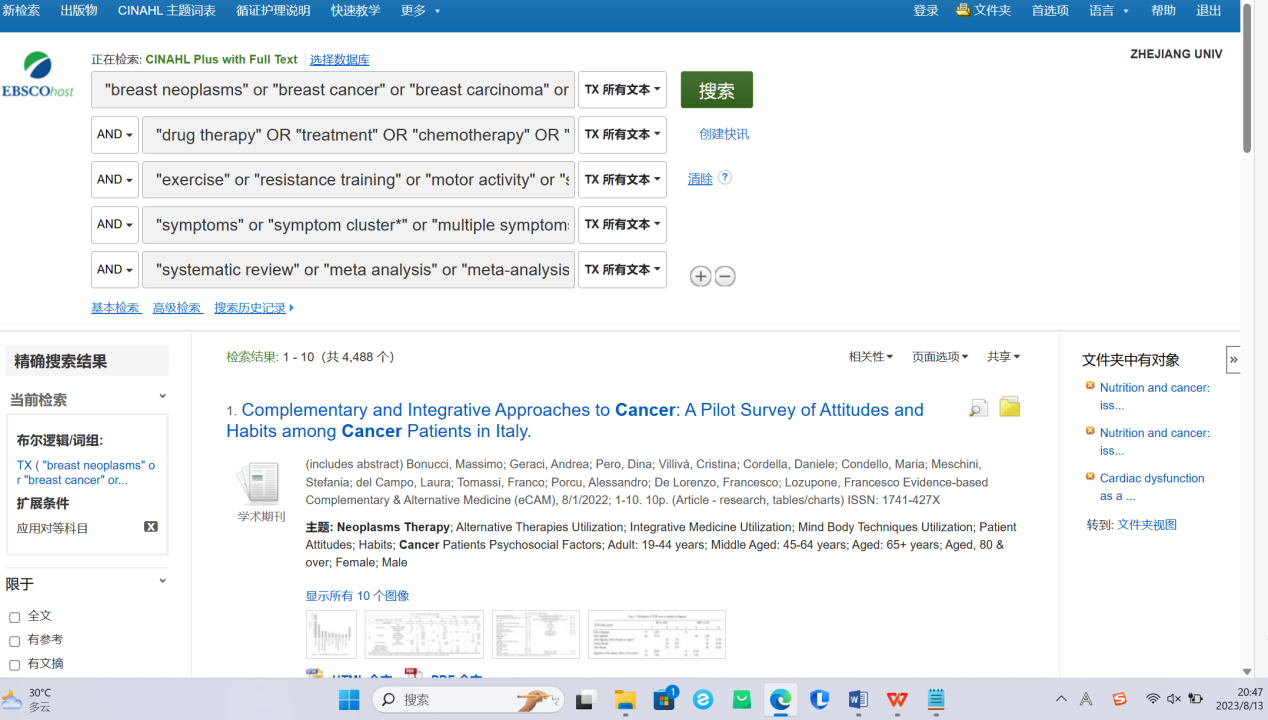


**PsycINFO 12**


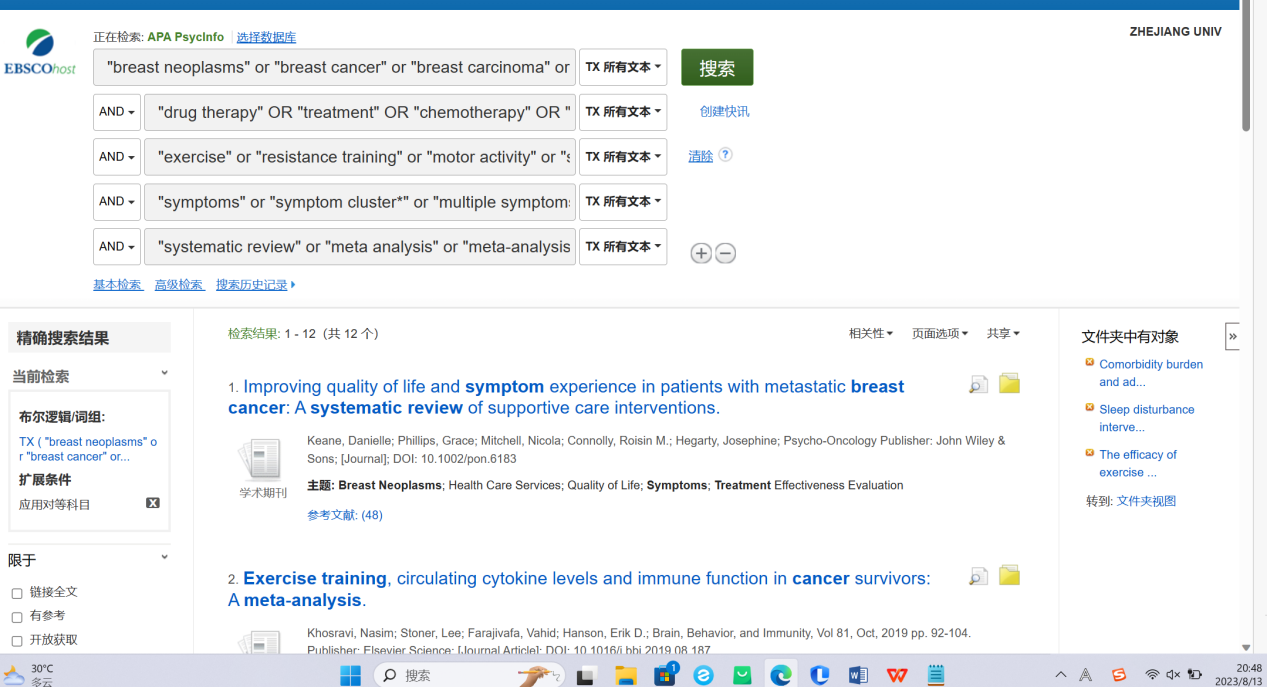


**Scopus**


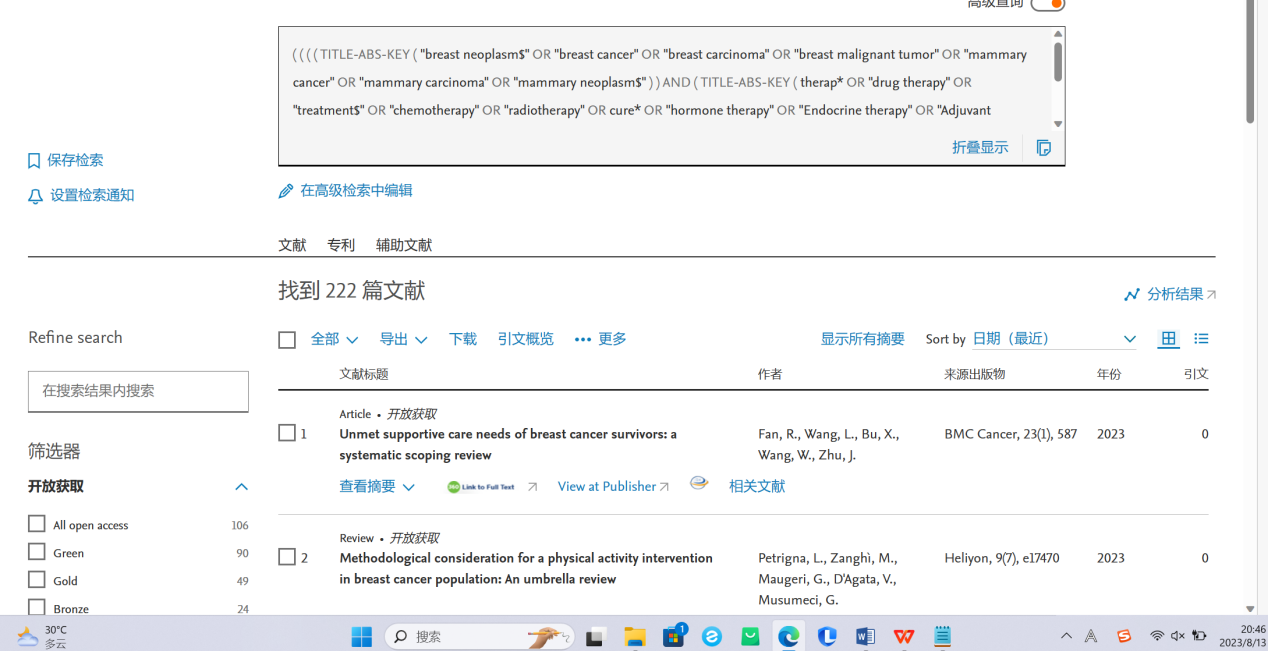


Wangfang data 19

主题:("乳腺癌" or "乳腺恶性肿瘤" or "乳腺肿瘤" or "乳癌") and 主题:(治疗 or 化疗 or "化学疗法" or 放疗 or "激素治疗" or "内分泌治疗" or "辅助治疗") and 主题:("症状群" or "症状簇" or "症状集" or 症状 ) and 主题:(运动 or 训练 or 锻炼 or 活动 or 行动 or 动身 or 行为 or 舞蹈 or 跳舞 or 瑜伽 or 走路 or 行走 or 步行 or 行进) and 全部:("系统评价" or "系统综述" or meta分析 or 荟萃分析 or “元分析”)


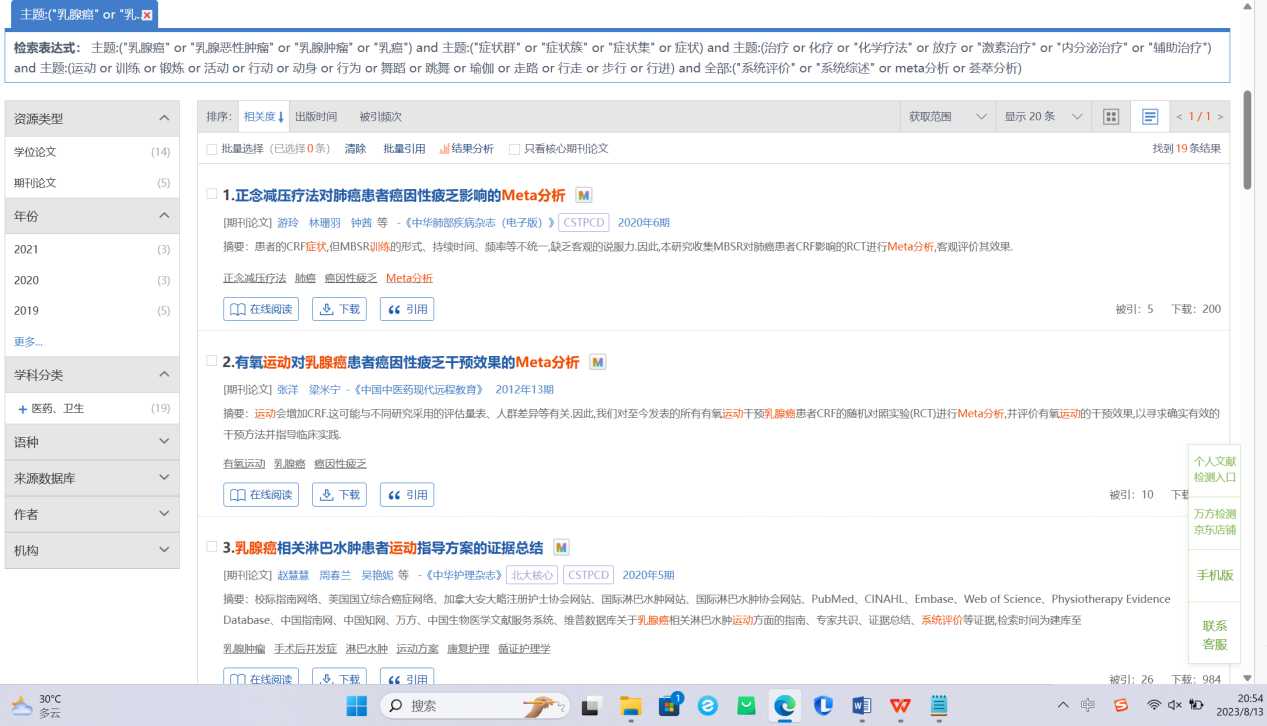


CNKI 7

"乳腺癌" + "乳腺恶性肿瘤" + "乳腺肿瘤" + "乳癌"

治疗 + 化疗 + "化学疗法" + 放疗 + "激素治疗" + "内分泌治疗" + "辅助治疗"

运动 + 训练 + 锻炼 + 活动 + 行动 + 动身 + 行为 + 舞蹈 + 跳舞 + 瑜伽 + 走路 + 行走 + 步行 + 行进

"症状群" + "症状簇" + "症状集" + 症状

"系统评价" + "系统综述" + meta分析 + 荟萃分析 + 元分析


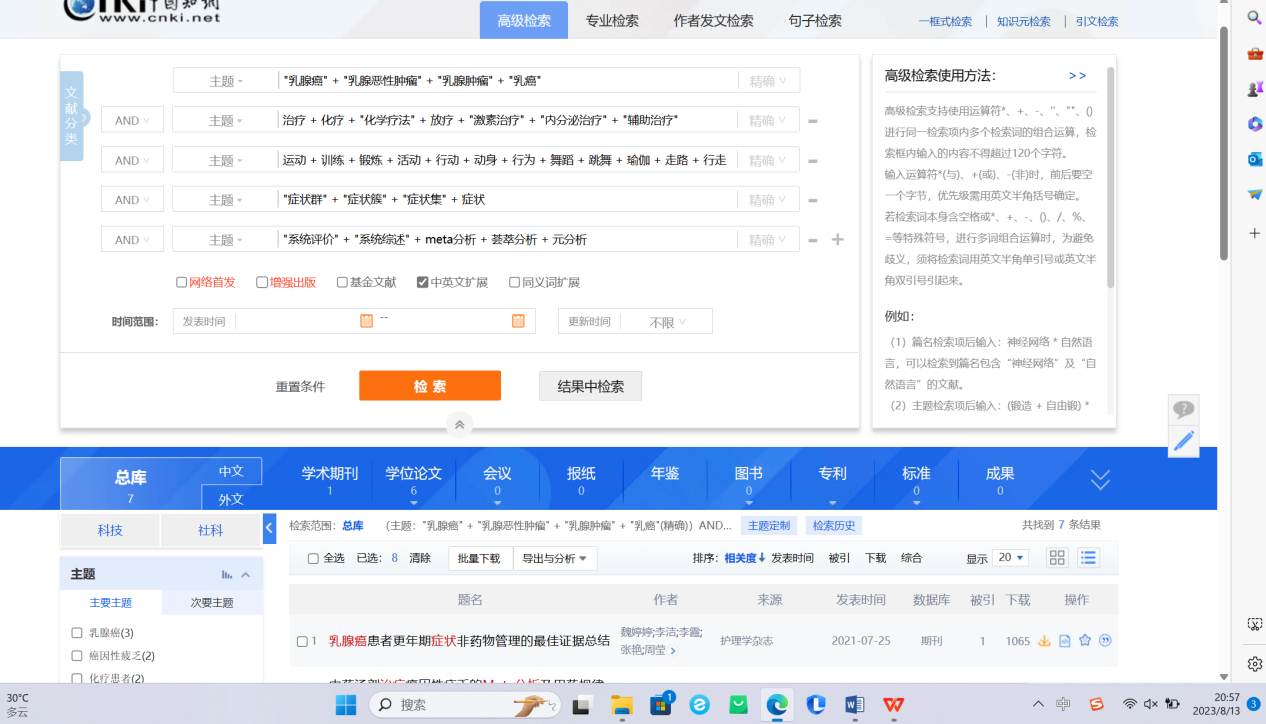


VIP 7

"乳腺癌" + "乳腺恶性肿瘤" + "乳腺肿瘤" + "乳癌"

治疗 + 化疗 + "化学疗法" + 放疗 + "激素治疗" + "内分泌治疗" + "辅助治疗"

运动 + 训练 + 锻炼 + 活动 + 行动 + 动身 + 行为 + 舞蹈 + 跳舞 + 瑜伽 + 走路 + 行走 + 步行 + 行进

"症状群" + "症状簇" + "症状集" + 症状

"系统评价" + "系统综述" + meta分析 + 荟萃分析 + 元分析


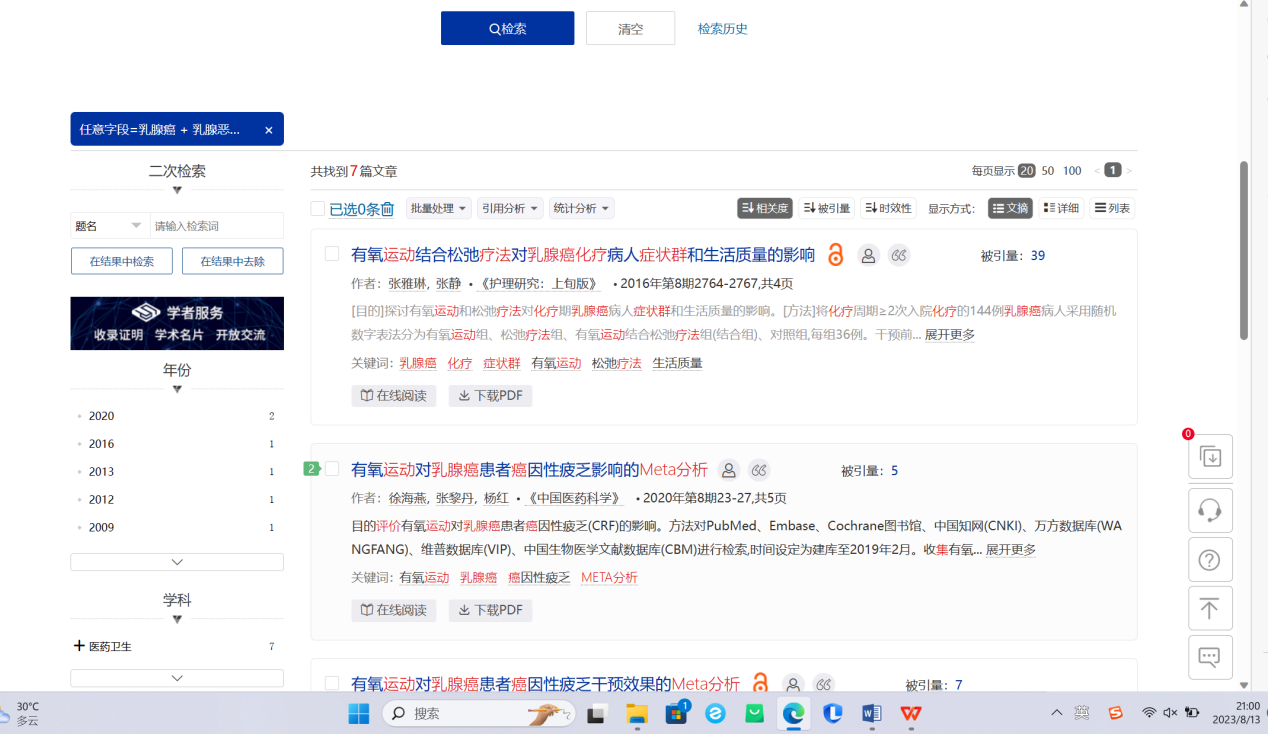


CBM (Sinomed) 11

( "乳腺癌"[全部字段:智能] OR "乳腺恶性肿瘤"[全部字段:智能] OR "乳腺肿瘤"[全部字段:智能] OR "乳癌"[全部字段:智能]) AND( "治疗"[全部字段:智能] OR "化疗"[全部字段:智能] OR "化学疗法"[全部字段:智能] OR "放疗"[全部字段:智能] OR "激素治疗"[全部字段:智能] OR "内分泌治疗"[全部字段:智能] OR "辅助治疗"[全部字段:智能]) AND( "症状群"[常用字段:智能] OR "症状簇"[常用字段:智能] OR "症状集"[常用字段:智能] OR "症状"[常用字段:智能]) AND( "运动"[常用字段:智能] OR "训练"[常用字段:智能] OR "锻炼 OR活动"[常用字段:智能] OR "行动"[常用字段:智能] OR "动身"[常用字段:智能] OR "行为"[常用字段:智能] OR "舞蹈"[常用字段:智能] OR "跳舞"[常用字段:智能] OR "瑜伽"[常用字段:智能] OR "走路"[常用字段:智能] OR "行走"[常用字段:智能] OR "步行"[常用字段:智能] OR "行进"[常用字段:智能]) AND( "系统评价"[常用字段:智能] OR "系统综述"[常用字段:智能] OR "meta分析"[常用字段:智能] OR "荟萃分析"[常用字段:智能])


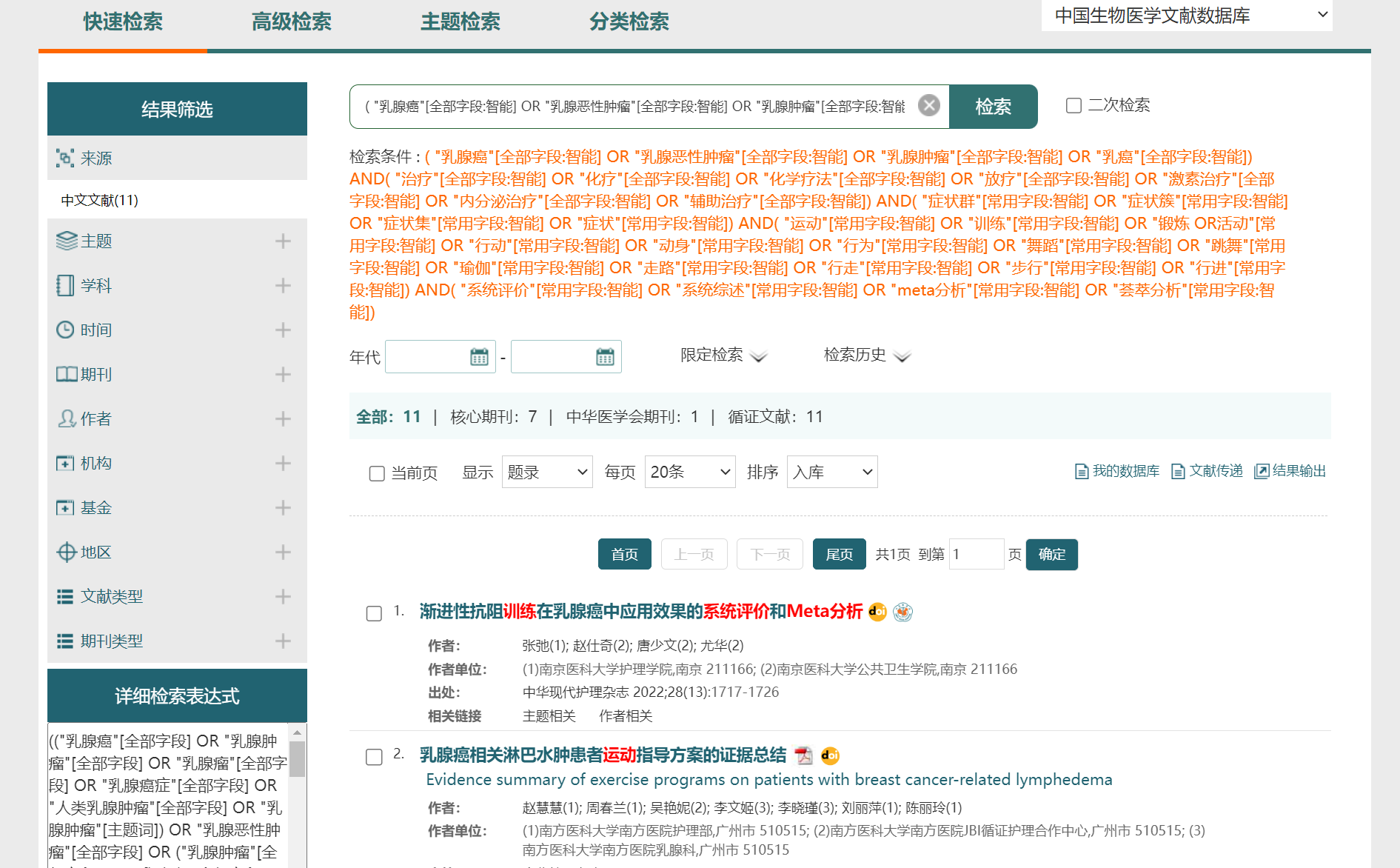

Supplement: Supplementary file 1 [file DataSheet_1.docx]
