# Supplementary material for: The effectiveness of exercise on the symptoms in breast cancer patients undergoing adjuvant treatment: an umbrella review of systematic reviews and meta-analyses
Source: Front Oncol. 2023 Sep 20;13:1222947. doi: 10.3389/fonc.2023.1222947 (PMC10548878; doi:10.3389/fonc.2023.1222947)
Supplement: Supplementary file 4 [file Table_3.docx]

**Chinese Databases Search Strategies Translation**

| **Databases** | **Search Strategies** | **Translation** |
| --- | --- | --- |
| Wanfang Data | 主题:("乳腺癌" or "乳腺恶性肿瘤" or "乳腺肿瘤" or "乳癌") and 主题:(治疗 or 化疗 or "化学疗法" or 放疗 or "激素治疗" or "内分泌治疗" or "辅助治疗") and 主题:("症状群" or "症状簇" or "症状集" or 症状 ) and 主题:(运动 or 训练 or 锻炼 or 活动 or 行动 or 动身 or 行为 or 舞蹈 or 跳舞 or 瑜伽 or 走路 or 行走 or 步行 or 行进) and 主题:("系统评价" or "系统综述" or meta分析 or 荟萃分析 or “元分析”) | Subject: (Breast Cancer or Breast Malignancy or Breast Tumor or Breast Carcinoma) and Subject: (Treatment or Chemotherapy or Radiation Therapy or Hormone Therapy or Endocrine Therapy or Adjuvant Therapy) and Subject: (Symptom Cluster or Symptoms) and Subject: (Exercise or Training or Workout or Activity or Mobility or Dancing or Yoga or Walking) and Subject: (Systematic Review or Meta-analysis ) |
| CNKI | 主题 ("乳腺癌" + "乳腺恶性肿瘤" + "乳腺肿瘤" + "乳癌") AND  主题 (治疗 + 化疗 + "化学疗法" + 放疗 + "激素治疗" + "内分泌治疗" + "辅助治疗") AND  主题 (运动 + 训练 + 锻炼 + 活动 + 行动 + 动身 + 行为 + 舞蹈 + 跳舞 + 瑜伽 + 走路 + 行走 + 步行 + 行进) AND  主题 ("症状群" + "症状簇" + "症状集" + 症状) AND  主题 ("系统评价" + "系统综述" + meta分析 + 荟萃分析 + 元分析) | Subject: (Breast Cancer or Breast Malignancy or Breast Tumor or Breast Carcinoma) AND  Subject: (Treatment or Chemotherapy or Radiation Therapy or Hormone Therapy or Endocrine Therapy or Adjuvant Therapy) AND  Subject: (Exercise or Training or Workout or Activity or Mobility or Dancing or Yoga or Walking) AND  Subject: (Symptom Cluster or Symptoms) AND  Subject: (Systematic Review or Meta-analysis ) |
| **Databases** | **Search Strategies** | **Translation** |
| VIP | 任意字段 ("乳腺癌" + "乳腺恶性肿瘤" + "乳腺肿瘤" + "乳癌") AND  任意字段 (治疗 + 化疗 + "化学疗法" + 放疗 + "激素治疗" + "内分泌治疗" + "辅助治疗") AND  任意字段 (运动 + 训练 + 锻炼 + 活动 + 行动 + 动身 + 行为 + 舞蹈 + 跳舞 + 瑜伽 + 走路 + 行走 + 步行 + 行进) AND  任意字段 ("症状群" + "症状簇" + "症状集" + 症状) AND  任意字段 ("系统评价" + "系统综述" + meta分析 + 荟萃分析 + 元分析) | Any field: (Breast Cancer or Breast Malignancy or Breast Tumor or Breast Carcinoma) AND  Any field: (Treatment or Chemotherapy or Radiation Therapy or Hormone Therapy or Endocrine Therapy or Adjuvant Therapy) AND  Any field: (Exercise or Training or Workout or Activity or Mobility or Dancing or Yoga or Walking) AND  Any field: (Symptom Cluster or Symptoms) AND  Any field: (Systematic Review or Meta-analysis ) |
| CBM | ( "乳腺癌"[全部字段:智能] OR "乳腺恶性肿瘤"[全部字段:智能] OR "乳腺肿瘤"[全部字段:智能] OR "乳癌"[全部字段:智能]) AND( "治疗"[全部字段:智能] OR "化疗"[全部字段:智能] OR "化学疗法"[全部字段:智能] OR "放疗"[全部字段:智能] OR "激素治疗"[全部字段:智能] OR "内分泌治疗"[全部字段:智能] OR "辅助治疗"[全部字段:智能]) AND( "症状群"[常用字段:智能] OR "症状簇"[常用字段:智能] OR "症状集"[常用字段:智能] OR "症状"[常用字段:智能]) AND( "运动"[常用字段:智能] OR "训练"[常用字段:智能] OR "锻炼 OR活动"[常用字段:智能] OR "行动"[常用字段:智能] OR "动身"[常用字段:智能] OR "行为"[常用字段:智能] OR "舞蹈"[常用字段:智能] OR "跳舞"[常用字段:智能] OR "瑜伽"[常用字段:智能] OR "走路"[常用字段:智能] OR "行走"[常用字段:智能] OR "步行"[常用字段:智能] OR "行进"[常用字段:智能]) AND( "系统评价"[常用字段:智能] OR "系统综述"[常用字段:智能] OR "meta分析"[常用字段:智能] OR "荟萃分析"[常用字段:智能]) | ( Breast Cancer[all fields:smart] OR Breast Malignancy[all fields:smart] OR Breast Tumor[all fields:smart] OR Breast Carcinoma[all fields:smart]) AND  ( Treatment[all fields:smart] OR Chemotherapy[all fields:smart] OR Radiotherapy"[all fields:smart ] OR Hormone therapy[all fields:smart] OR Endocrine therapy[all fields:smart] OR Adjuvant therapy[all fields:smart]) AND  ( Symptom cluster[common field:smart] OR Symptoms[common field:smart]) AND  ( Exercise[ Common Fields:Intelligent] OR Training[Common Fields:Intelligent] OR Activity[Common Fields:Intelligent] OR Moving[Common Fields:Intelligent] OR Dancing[Common Fields:Intelligent] OR Yoga[Common Fields. Intelligent] OR Walking[Common Fields:Intelligent] AND  (Systematic Review[Common Fields:Intelligent] OR Meta-analysis[Common Fields:Intelligent] |

Note: Due to the existence of multiple translations of the word, the Chinese and English texts cannot be matched one to the other.

Translation of retrieved Chinese literature

| 中药汤剂治疗癌因性疲乏的Meta分析及用药规律挖掘 | Meta-analysis of Chinese herbal tonics in treating cancer-caused fatigue and mining of medication patterns |
| --- | --- |
| 正念冥想对血液肿瘤化疗患者癌因性疲乏及焦虑抑郁的应用研究 | Study on the Application of Positive Thought Meditation on Cancer-caused Fatigue and Anxiety and Depression in Hematologic Cancer Chemotherapy Patients |
| 正念减压疗法在乳腺癌患者中应用效果的Meta分析 | Meta-analysis of the effect of stress-reducing therapy in breast cancer patients. |
| 正念减压疗法对肺癌患者癌因性疲乏影响的Meta分析 | Meta-analysis of the effect of orthostatic stress reduction therapy on cancer-caused fatigue in lung cancer patients |
| 针刺临床证据整合评估及证据图谱的制订研究 | A study on the evaluation of the integration of clinical evidence and the development of evidence mapping of acupuncture. |
| 运动机能贴扎改善乳腺癌术后淋巴水肿疗效的系统评价 | Systematic Review of the Efficacy of Kinesio Taping to Improve Postoperative Lymphedema in Breast Cancer Patients |
| 瑜伽运动对乳腺癌患者负性情绪及生活质量影响的meta分析 | Meta-analysis of the Effects of Yoga Exercise on Negative Emotions and Quality of Life of Breast Cancer Patients |
| 有氧运动结合松弛疗法对乳腺癌化疗病人症状群和生活质量的影响 | Effects of aerobic exercise combined with relaxation therapy on symptom clusters and quality of life of breast cancer chemotherapy patients |
| 有氧运动对乳腺癌患者癌因性疲乏影响的Meta分析 | Meta-analysis of the effect of aerobic exercise on cancer-caused fatigue in breast cancer patients |
| 有氧运动对乳腺癌患者癌因性疲乏干预效果的Meta分析 | Meta-analysis of the effect of aerobic exercise on cancer-caused fatigue in breast cancer patients. |
| 延续性护理对乳腺癌病人焦虑抑郁及生活质量影响的系统评价 | A Systematic Review of the Effects of Extended Care on Anxiety, Depression, and Quality of Life in Breast Cancer Patients |
| 胃肠癌化疗患者应对方式的认知行为干预 | Cognitive Behavioral Intervention on Coping Styles of Gastrointestinal Cancer Chemotherapy Patients |
| 疏肝健脾法治疗乳腺癌的疗效评价及对瘤前抑郁障碍小鼠乳腺癌作用机制研究 | Evaluation of the efficacy of liver-sparing and spleen-strengthening method in the treatment of breast cancer and the mechanism of action on breast cancer in mice with pre-tumor depressive disorder |
| 疏肝调神针法治疗乳腺癌相关轻中度抑郁的临床研究 | Clinical study on the treatment of mild to moderate depression associated with breast cancer by liver-sparing and spleen-tuning acupuncture method |
| 乳腺癌抑郁中医证候系统综述 | A Review of Chinese Medical Evidence System for Breast Cancer Depression |
| 乳腺癌相关淋巴水肿患者运动指导方案的证据总结 | Evidence summary of exercise guidance programs for patients with breast cancer-associated lymphedema |
| 乳腺癌围手术期中西医结合临床护理路径的编制与应用研究 | A study on the compilation and application of combined Chinese and Western medicine clinical nursing pathways in the perioperative period of breast cancer |
| 乳腺癌术后患者功能康复训练效果的系统评价 | Systematic Review of the effect of functional rehabilitation training in postoperative breast cancer patients |
| 乳腺癌术后患者的症状困扰及其与生活质量的相关性 | Symptom distress and its correlation with quality of life in postoperative breast cancer patients |
| 乳腺癌患者内分泌治疗服药依从性现况及护士主导的个体化随访干预方案构建研究 | Study on the current status of endocrine therapy medication adherence of breast cancer patients and the construction of nurse-led individualized follow-up intervention program |
| 乳腺癌患者更年期症状非药物管理的最佳证据总结 | Summary of the best evidence for non-pharmacological management of menopausal symptoms in breast cancer patients |
| 康复护理干预对乳腺癌患者上肢水肿及生命质量影响的Meta分析 | Meta-analysis of the effects of rehabilitation nursing interventions on upper extremity edema and quality of life in breast cancer patients |
| 聚焦解决模式对乳腺癌围术期患者焦虑抑郁症状影响的Meta分析 | Meta-analysis of the effects of the Focused Solution Model on anxiety and depressive symptoms in perioperative breast cancer patients |
| 渐进性抗阻训练在乳腺癌中应用效果的系统评价和Meta分析 | Systematic Review and Meta-analysis of the Effectiveness of Progressive Resistance Training in Breast Cancer. |
| 基于文献的蜂针临床治疗病谱研究及Meta分析 | Literature-based Study and Meta-analysis of Clinical Therapeutic Disease Spectrum of Bee Acupuncture |
| 基于JBI循证模式的乳腺癌淋巴水肿管理证据转化研究 | Translational Study of Evidence for the Management of Lymphedema in Breast Cancer Based on the JBI Evidence-Based Model |
| 钙稳态失衡对钙记忆相关蛋白表达的影响机制及钙离子调节剂临床疗效的荟萃分析 | Mechanism of calcium homeostasis imbalance on calcium memory-related protein expression and meta-analysis of clinical efficacy of calcium ion modifiers |
| 钙及维生素D对早期乳腺癌患者骨量的影响 | Effect of calcium and vitamin D on bone mass in patients with early-stage breast cancer |
| 非药物补充替代医学辅助治疗乳腺癌术后不良反应的循证研究 | Evidence-based study of non-pharmacologic complementary alternative medicine in the adjuvant treatment of postoperative adverse effects of breast cancer |
| 放化疗对乳腺癌妇女性身份和性功能影响的多因素研究 | A multifactorial study on the effects of radiotherapy on sexual identity and function in women with breast cancer |
| 补肾促卵方治疗多囊卵巢综合征导致排卵障碍性不孕症的临床研究 | Clinical study on the treatment of ovulation disorder infertility due to polycystic ovary syndrome by tonifying kidney and promoting ovulation formula |
| TRPS1在胃癌中的表达及对胃癌细胞增殖、迁移和侵袭作用的研究 | Study on the expression of TRPS1 in gastric cancer and its effect on the proliferation, migration and invasion of gastric cancer cells |
| NBS1基因多态性与慢性乙肝病毒感染引起的肝细胞癌的相关性及生物学功能研究 | Correlation and biological function of NBS1 gene polymorphisms in hepatocellular carcinoma caused by chronic hepatitis B virus infection |
| MT2--MMP在肾透明细胞癌中的表达及其在肿瘤增殖和侵袭中的作用机制研究 | MT2 - Expression of MMP in renal clear cell carcinoma and its role in tumor proliferation and invasion |
| Her-2表达与食管癌预后关系的Meta分析 | Meta-analysis of the relationship between Her-2 expression and prognosis of esophageal cancer |
